# Image Encryption Scheme Based on Multiscale Block Compressed Sensing and Markov Model

**DOI:** 10.3390/e23101297

**Published:** 2021-09-30

**Authors:** Yuandi Shi, Yinan Hu, Bin Wang

**Affiliations:** The Key Laboratory of Advanced Design and Intelligent Computing, Ministry of Education, School of Software Engineering, Dalian University, Dalian 116622, China; syd35555@gmail.com (Y.S.); huyinan@dlu.edu.cn (Y.H.)

**Keywords:** image encryption, multiscale block compressed sensing, state transition matrix, Markov model

## Abstract

Many image encryption schemes based on compressed sensing have the problem of poor quality of decrypted images. To deal with this problem, this paper develops an image encryption scheme by multiscale block compressed sensing. The image is decomposed by a three-level wavelet transform, and the sampling rates of coefficient matrices at all levels are calculated according to multiscale block compressed sensing theory and the given compression ratio. The first round of permutation is performed on the internal elements of the coefficient matrices at all levels. Then the coefficient matrix is compressed and combined. The second round of permutation is performed on the combined matrix based on the state transition matrix. Independent diffusion and forward-backward diffusion between pixels are used to obtain the final cipher image. Different sampling rates are set by considering the difference of information between an image’s low- and high-frequency parts. Therefore, the reconstruction quality of the decrypted image is better than that of other schemes, which set one sampling rate on an entire image. The proposed scheme takes full advantage of the randomness of the Markov model and shows an excellent encryption effect to resist various attacks.

## 1. Introduction

With the rapid development of internet technology, digital images are widely used in all walks of life. As images can intuitively display information, they are widely used in banking, the military, medicine, finance, and other fields. Once images containing important information spread on the internet, they are likely to be attacked and cracked by hackers. To make meaningful images meaningless through encryption can effectively protect the security of private information in transmission and storage.

Technology applied to image encryption includes chaotic systems [1,2,3,4,5,6,7,8], cellular automatons [9,10,11], substitution boxes [12,13,14], DNA encoding [15,16,17,18,19,20], elliptic curves [21,22,23,24], finite-precision error [25], Galois field [26] and quantum computing [27,28]. The chaotic system is most popular in the field of image encryption and is continuously improved, such as symmetric chaotic maps [29] and adaptive chaotic maps [30,31]. In particular, the improvement of the traditional one-dimensional chaotic system [1,2,3,12,32] enhances the encryption effect. Therefore, we choose several chaotic systems with excellent encryption effects to generate chaotic sequences.

The combination of compressed sensing (CS) theory and image encryption algorithms, by which an image is simultaneously compressed and encrypted, has seen much research. The most special advantage of compressed sensing is that it can reduce data redundancy. Before data transmission, if the cipher image can be further compressed, it will not only improve the transmission efficiency but also be more suitable for transmission channels with limited bandwidth. Moreover, because the sampling process of compressed sensing is a linear random projection process, in the presence of noise, the target sparse signal can be reconstructed with high probability through a greedy algorithm or an optimized algorithm. This not only protects the security of information but saves bandwidth, time, and storage space. There are both one-dimensional (1D) [33,34,35,36] and two-dimensional (2D) compressed sensing [37], and encryption schemes for both single [33,38] and multiple images [39,40]. Chai et al. [39] proposed a scheme to compress and encrypt two color images at the same time through parallel operations on their RGB components, which improves efficiency and enhances security. To further reduce the bandwidth and computing load, Fan et al. [41] proposed an algorithm combined with vector quantization (VQ) and CS, distinguishing important and secondary information. Important data are extracted by a VQ compression algorithm, and the secondary data are compressed by a CS algorithm, so as to achieve higher compression efficiency. However, many encryption schemes are generated based on traditional compressed sensing theory, using one sampling rate for the whole image and a single measurement matrix to measure it, which is neither efficient nor safe enough [33,34,35,36,38,42]. Additionally, when the compression ratio is low, the reconstructed image after decryption has a poor visual effect.

Many effective encryption schemes with improved efficiency have been proposed. Wang et al. [43] proposed a scheme based on parallel compressive sensing (PCS) combined with count mode, where the measurement matrix is updated by continuously updating the key values. At the same time, the sampling object is reduced from the whole image matrix to a single column vector. Therefore, this scheme cannot only improve the efficiency but can effectively resist the chosen plain attack (CPA). Wen et al. [40] reduced the scale of single sampling by applying the semi-tensor product (STP) to the process of compressed sensing, employing the STP strategy for the sparse matrix of the plain image and measurement matrix, and cascading multiple images after compression into the main image for image encryption, reducing the storage scale of the measurement matrix and data transmission. To reduce the computation scale and improve security, Zhu et al. [44] designed the BCS-CRP framework, which divides the image coefficient matrices obtained by wavelet transform into blocks, using the coefficient random permutation (CRP) strategy to confuse each coefficient vector, with a good encryption effect according to simulation results.

The quality of a reconstructed image is improved mainly from two aspects. One aspect is to change the method of wavelet decomposition and image reconstruction. Chai et al. [33] designed a contrast experiment by combining different wavelet decomposition methods two-dimensional discrete cosine transform (DCT2), discrete wavelet transform (DWT) and different reconstruction algorithms smoothed l0 norm (SL0), orthogonal matching pursuit (OMP) to encrypt and decrypt an image. Experimental results showed that the adoption of DWT was more helpful for decryption. The other aspect is to optimize the measurement matrix through singular value decomposition (SVD) and optimization. The modified measurement matrix can satisfy the RIP condition of compressed sensing theory with high probability, which greatly improves the reconstruction quality of the decrypted image. Chai et al. [39] designed an algorithm to optimize the measurement matrix by SVD, and verified by simulation experiments that using the optimized measurement matrix to measure the image, the final decrypted image quality is better than without optimization.

Many encryption schemes fail to fully consider the information distribution characteristics of natural images, resulting in poor reconstruction quality of decrypted images. Gan et al. [10] used a circular matrix to construct the measurement matrix, with only one sample rate set for sampling the whole image matrix in each encryption process. From their experimental results, the values of the peak signal to noise ratio (PSNR) between the plain images and corresponding decrypted images at different sampling rates were not high enough, and as the sampling rate increased, the increase in PSNR was not large. Luo et al. [28] decomposed the image matrix into four coefficient matrices firstly, then retained the low-frequency matrix and used two measurement matrices generated by two different sampling rates to compress the remaining coefficient matrices separately. Their proposed scheme obtained the decrypted image with higher reconstruction quality under the same compression ratio. Based on the premise that different sampling rates should be set for coefficient matrices of different frequencies, we apply the theory of multiscale block compressed sensing to the design of an image encryption scheme. The low-frequency coefficient matrix of an image is fully sampled, and different sampling rates are set for the remaining coefficient matrices according to the amount of information they carry. There is a significant improvement in the reconstructed image after decryption. Different from the traditional encryption algorithms like DNA coding [15,16], cellular automata [9,10], and substitution box [12,13] that follow generally known rules to carry out the subsequent work, this paper introduces the Markov model in machine learning, and the scrambling process is carried out according to the information of the plain image and chaotic sequences. Experimental results show that the proposed scheme achieves a good encryption effect.

Our contributions are as follows.

1. An encryption architecture of permutation, compression, secondary scrambling, and diffusion is designed, which shows good compression performance and guarantees security;
2. A transition probability matrix in a Markov model is introduced to scramble the image and define the state space according to the characteristics of image pixel values in the encryption process. The state transition probability matrix is constructed based on the distribution of pixel values. The process achieves good randomness, so it is difficult to predict;
3. Information about plain images and chaotic sequences is used in the encryption process, giving the scheme high plain sensitivity to resist known-plaintext attacks (KPAs) and chosen-plaintext attacks (CPAs);
4. Multiscale block compressed sensing theory is introduced, sampling rates of images are set by a more reasonable approach, and the reconstruction quality of decrypted images is greatly improved.

The rest of this paper is organized as follows. Preliminaries are discussed in Section 2. The steps of the proposed scheme are described in Section 3. Simulation results are shown in Section 4, and a sensitivity analysis of the scheme is discussed in Section 5. Conclusions are drawn in Section 6.

## 2. Materials and Methods

### 2.1. Multiscale Block Compressed Sensing

Fowler et al. [45] proposed a multiscale block compressed sensing algorithm based on a wavelet domain. The MS-BCS-SPL algorithm divides the image into blocks based on the BCS algorithm and samples each image sub-block with a different matrix. The original image is decomposed by a multilayer wavelet transform, and the wavelet coefficients of each layer are divided into blocks whose size varies with the number of layers. A measurement matrix, determined by the sampling rate of each layer, is used for measurement. Since the different levels of wavelet decomposition have different importance to the final image reconstruction quality, each layer corresponds to a different sampling rate. The smooth projection Landweber method is used to reconstruct each image block; thus, the complete reconstructed image can be obtained.

As the main information of an image is concentrated in the low-frequency coefficient after wavelet decomposition, its details are concentrated in high-frequency coefficients. To improve the reconstruction quality of an image requires one to keep the low-frequency part of the image and abandon the high-frequency part as much as possible. The measurement matrix A of the original image is decomposed into multiscale transformation matrix Ω and multiscale measurement matrix $\Phi^{\prime }$, and *A* is represented by $A=\Phi^{\prime }\Omega$, so the compression process can be expressed as

(1) $$y=Ax=\Phi^{\prime }\Omega x$$

The multiscale transformation matrix Ω is decomposed by an L-level wavelet transform to form *L* measurement operators, which constitute the multiscale block measurement matrix $\Phi^{\prime }$. The wavelet decomposition process of the original image *x* can be expressed as

(2) $$\tilde{x}=\Omega x$$

The *s*-th sub-band of $\tilde{x}$ is cut into sub-blocks of size $B_{l}\times B_{l}$, each sampled by a sampling matrix of corresponding size. For example, the process of compressing the *j*-th sub block can be expressed as

(3) $$y_{l,s,j}=\Phi_{l}\tilde{x},\;s\in \;\left\{H,V,D\right\},\;1\leq l\leq L$$

After the original image is decomposed, the influence of the decomposition block of each layer on the image reconstruction is different. To improve the reconstruction quality of the image, it is necessary to set the corresponding sampling rate for each wavelet decomposition layer. Let the baseband sampling rate of wavelet decomposition be 1, so $S_{0}=1$, and the wavelet decomposition sub-rate of each layer can be expressed as

(4) $$S_{l}=W_{l}S^{\prime }$$

where $W_{l}$ is the weight of each layer after wavelet decomposition in the whole image, i.e.,

(5) $$W_{l}=16^{L-l+1}$$

For the whole image, the sampling rate can be expressed as

(6) $$S=\frac{1}{4^{L}}S_{0}+\sum_{l=1}^{L}\frac{3}{4^{L-l+1}}W_{l}S^{\prime }$$

If the sampling rate S of the whole image and the weight $W_{l}$ of each wavelet decomposition layer are known, then the total sampling rate $S^{\prime }$ can be calculated by Equation (6). The sampling rate $S_{l}$ of the *l*-th level wavelet decomposition coefficient can be obtained by substituting $S^{\prime }$ in Equation (4). When solving for the sampling rate of each layer after wavelet decomposition, there will be multiple solutions greater than 1. In practice, the sampling rate of each layer should not be greater than 1, so such solutions should be set to 1. In this case, the sampling rate is expressed as

(7) $$S=\frac{1}{4^{L}}S_{0}+\frac{3}{4^{L}}S_{1}+\sum_{l=2}^{L}\frac{3}{4^{L-l+1}}W_{l}S^{\prime }$$

We recalculate $S_{l}$ from Equation (7). The above process is repeated for the wavelet decomposition of each layer l=2,3,…,L, and we check for $S_{l}>1$ to ensure that $S_{l}\leq 1$ in each layer.

### 2.2. Chaotic Systems

The tent-logistic-tent system (TLTS) and tent-sine-tent system (TSTS) are, respectively, obtained as [32]

(8) $$X_{n+1}={\;f}_{\mathrm{TLT}}\left(X_{n},r\right)\;=\;\left\{\begin{array}{l} \left(\frac{r^{2}}{2}X_{n}\left(1-\frac{r}{2}X_{n}\right)+\frac{r}{2}X_{n}\right)r^{14}\mathrm{mod}1,X_{n}<0.5\\ \left(\left(\frac{r^{2}}{2}\right.\left(1-X_{n}\right)\left(1-\frac{r}{2}\left(1-X_{n}\right)\right)+\left.\frac{r}{2}\left(1-X_{n}\right)\right)r^{14}\right)\mathrm{mod}1,X_{n}\geq 0.5 \end{array}\right.$$

(9) $$X_{n+1}={\;f}_{\mathrm{TST}}\left(X_{n},r\right)\;=\;\left\{\begin{array}{c} \left(\frac{r}{4}\sin\left(\pi \frac{r}{2}X_{n}\right)+\frac{r}{2}X_{n}\right)r^{14}\mathrm{mod}1,X_{n}<0.5\\ \left(\left(\frac{r}{4}\sin\left(\pi \frac{r}{2}\left(1-X_{n}\right)\right)\right.+\left.\frac{r}{2}\left(1-X_{n}\right)\right)r^{14}\right)\mathrm{mod}1,X_{n}\geq 0.5 \end{array}\right.$$

where $r\in \;\left(1.05.4\right]$ is the control parameter of the TLT and TST chaotic systems. When r is greater than 1.05, the LE value of the chaotic system is positive, i.e., the system is in a chaotic state.

The hybrid chaotic system [12] is defined as

(10) $$H=\;\left\{\begin{array}{c} \left(\left(r^{10}\right)\frac{r}{4}\sin\left(\pi \frac{r^{2}}{2}x_{i}\right)\left(1-x_{i}\right)\right)\mathrm{mod}1,{\mathrm{rx}}_{i}\left(1-x_{i}\right)\;\leq \frac{1}{2}\\ \left(\left(r^{10}\right)\frac{r}{4}\sin\left(\pi \frac{r}{2}\left(1-{\mathrm{rx}}_{i}\left(1-x_{i}\right)\right)\right)\right)\mathrm{mod}1,{\mathrm{rx}}_{i}\left(1-x_{i}\right)\;>\frac{1}{2} \end{array}\right.$$

where $r\in \;\left[1.4,4\right]$ is the control parameter of the hybrid chaotic map. When r is greater than 1.1, the LE value of the chaotic system is positive, i.e., the system is in a chaotic state.

We use the three chaotic systems to generate three measurement matrices, respectively.

A new chaotic system-improved sine-exponent-logistic(ISEL) is obtained as [46]

(11) $$X_{n+1}={\left(\sin\left({\pi X}_{n}\right)\right)}^{a\ln\left(4{\mathrm{bX}}_{n}\left(1-X_{n}\right)+c\right)}$$

where $a\in \;\left[0,1\right]$, $b\in \;\left[0,5\right]$, $c\in \;\left[1.5,2.8\right]$ are the control parameters of the ISEL system. We use this system to generate chaotic sequences for diffusion.

### 2.3. Markov Model

The Markov model can be used to simulate random processes [47]. In our scheme, a Markov chain is used to construct the state transition matrix for scrambling the image matrix.

#### 2.3.1. State Space

In the Markov chain, every variable has several possible values, and the set of all these is called the state space. To generate this, numbers are divided into four categories in our scheme:

If the integer part of a number is positive and odd, then it is a positive-odd (po-od) number;

If the integer part of a number is negative and odd, then it is a negative-odd (ne-od) number;

If the integer part of a number is positive and even, then it is a positive-even (po-ev) number;

If the integer part of a number is negative and even, then it is a negative-even (ne-ev) number.

#### 2.3.2. Markov State Transition Matrix

Obviously, since there is more than one state at each time, there are several cases of transferring from the previous to the current state. All conditional probabilities form a transition probability matrix, as shown in Table 1. Here, $a_{i},i=1,2,\ldots$ is the state at the previous time, $a_{j},j=1,2,\ldots$ is the state at the current time, and $p_{ij},i=1,2,\ldots ;j=1,2,\ldots$ is the conditional probability from $a_{i}$ to $a_{j}$.

According to the above definition, the header of the state transition matrix is constructed.

Next, we initialize a matrix f of size 4 × 4, which is used to record the frequency of state transition. For each column vector of the matrix to be measured, we first determine the type of the first element, i.e., the row coordinate of the state transition matrix. Then the type of the next element is determined, i.e., the column coordinate of the state transition matrix. The position in the matrix f corresponding to the coordinate point is incremented by 1 to obtain the updated matrix f. For example, the matrix to record the frequency of state transition is shown in Table 2. We calculate the sum of four frequencies in each row of the matrix f, respectively, which means the total frequency of transitions from one type of number to other types of number. Each element is divided by the sum of the corresponding row to get a probability, which means the transition probability from one type of number to another type of number. After all probabilities are calculated, the row-based state transition probability matrix (RSTPM) is obtained, as shown in Table 3. The generation process of the column-based state transition probability matrix (CSTPM) is similar to that of RSTPM, where we get a row vector instead of a column vector of the matrix.

For RSTPM, we find all positions of the probabilities greater than 0.25 of the matrix shown in Table 3. The row coordinates indicate whether odd- or even-column vectors are to be selected, and whether the direction of movement is up or down. If the row coordinate is odd (even), then odd (even)-column vectors are selected; if the row coordinate is positive (negative), then all selected elements move up (down). Specifically, if the row coordinate is a po-od (ne-od) number, then all odd-column vectors move up (down), and if the row coordinate is a po-ev (ne-ev) number, then all even-column vectors move up (down). The column coordinates indicate the number of cyclic shifts, whose values are generated by the chaotic map.

For CSTPM, we find all positions of the probabilities greater than 0.25 of the matrix like Table 3. The row coordinates indicate whether odd- or even-row vectors are to be selected, and whether the direction of movement is left or right. If the row coordinate is odd (even), then the odd (even)-row vectors are selected, and if the row coordinate is positive (negative), then all selected elements move left (right). Specifically, if the row coordinate is a po-od (ne-od) number, then all odd-row vectors move left (right), and if the row coordinate is a po-ev (ne-ev) number, then all even-row vectors move left (right). The column coordinates indicate the number of cyclic shifts, whose values are generated by the chaotic map.

## 3. Scheme Based on Multiscale Block Compressed Sensing

**Figure 1:**
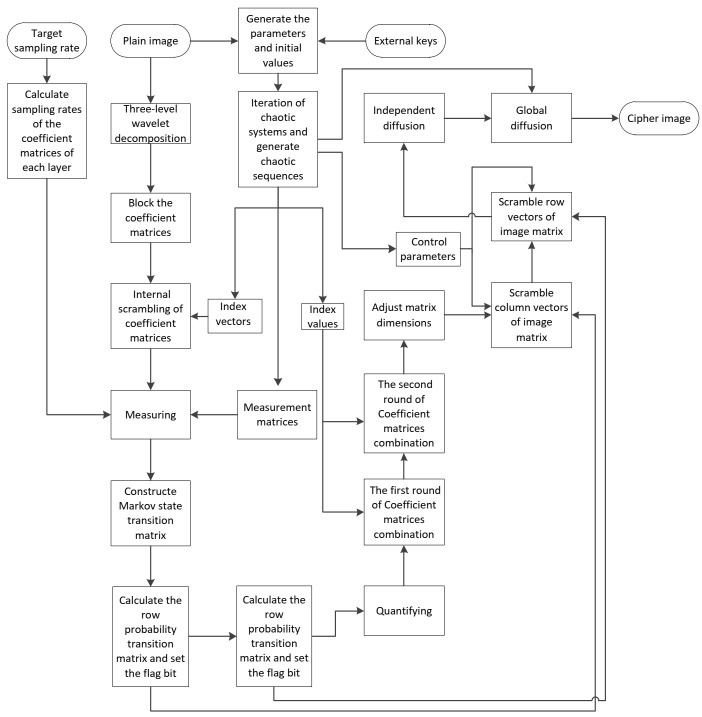
Flowchart of image encryption process.

The proposed encryption scheme is shown in Figure 1. The image is decomposed by discrete wavelet transform to get the coefficient matrices, and each coefficient matrix is scrambled for the first time and measured by the corresponding measurement matrix. After compression, all matrices of measurement values are merged, and that matrix is scrambled by state transition matrices. The final cipher image is obtained by diffusion.

### 3.1. Encryption Process

#### 3.1.1. Generating Parameters and Initial Values for Chaotic System

The encryption process should be closely related to plain information to make it resistant to known- and chosen-plaintext attacks. The hash value K of the plain image is generated by the SHA256 function. K is converted to binary numbers, and 32 groups of these are generated by dividing every eight bits into a group,

(12) $$K\;={\;k}_{1},k_{2},\ldots ,k_{32}$$

We count the number of zeros, number L of ones, length of the longest continuous zero sequence, and length of the longest continuous ones sequence in the hash value K as $L_{0}$, $L_{1}$, $l_{0}$, and $l_{1}$, respectively, and

(13) $$r_{0}=\;\left\{\begin{array}{l} 4-\frac{\mathrm{mod}\left(\left(\frac{l_{0}}{L_{0}}+\frac{l_{1}}{L_{1}}\right)\times 40,1\right)}{10},\left(\frac{l_{0}}{L_{0}}+\frac{l_{1}}{L_{1}}\right)\times 40>4\\ \left(\frac{l_{0}}{L_{0}}+\frac{l_{1}}{L_{1}}\right)\times 40,\left(\frac{l_{0}}{L_{0}}+\frac{l_{1}}{L_{1}}\right)\times 40\leq 4 \end{array}\right.$$

(14) $$\left\{\begin{array}{l} r_{1}=1.05+\mathrm{mod}\left(\left(\frac{\left(\max\left(k_{1},k_{2},k_{3},k_{4},k_{5},k_{6}\right)\times t_{1}\right)}{\left(\min\left(k_{1},k_{2},k_{3},k_{4},k_{5},k_{6}\right)\times t2\right)}\right)\times 10^{14},2.95\right)\\ r_{2}=1.05+\mathrm{mod}\left(\left(\frac{\left(\max\left(k_{7},k_{8},k_{9},k_{10},k_{11},k_{12}\right)\times t_{3}\right)}{\left(\min\left(k_{7},k_{8},k_{9},k_{10},k_{11},k_{12}\right)\times t4\right)}\right)\times 10^{14},2.95\right) \end{array}\right.$$

(15) $$\left\{\begin{array}{l} a\;=1.0/\left(1+e^{\frac{1}{256}\times \left(-t_{5}\times \left(k_{13}⊕k_{14}⊕k_{15}\right)\right)}\right)\\ b\;=5.0/\left(1+e^{\frac{1}{256}\times \left(-t_{6}\times \left(k_{16}⊕k_{17}⊕k_{18}\right)\right)}\right)\\ c\;=\frac{1}{2}\times \left(1.5/\left(1+e^{\frac{1}{256}\times \left(-t_{7}\times \left(k_{19}⊕k_{20}⊕k_{21}\right)\right)}\right)+2.8/\left(1+e^{\frac{1}{256}\times \left(-t_{8}\times \left(k_{22}⊕k_{23}⊕k_{24}\right)\right)}\right)\right) \end{array}\right.$$

We construct

(16) $$\left\{\begin{array}{l} Z_{1}=\;\left[\begin{array}{cc} t_{1}\times k_{25} & t_{3}\times k_{26}\\ t_{5}\times k_{27} & t_{7}\times k_{28} \end{array}\right]\\ Z_{2}=\;\left[\begin{array}{cc} t_{2}\times k_{29} & t_{4}\times k_{30}\\ t_{6}\times k_{31} & t_{8}\times k_{32} \end{array}\right] \end{array}\right.$$

and find their Kronecker product,

(17) $$Z_{3}=\;\left(Z_{1}\right)⊗\left(Z_{2}\right)$$

where ⊗ represents the Kronecker product operator,

(18) $$\left\{\begin{array}{c} x_{0}=\mathrm{mod}\left(\left(Z_{3}\left(1\right)+Z_{3}\left(2\right)-\left\lfloor Z_{3}\left(1\right)+Z_{3}\left(2\right)\right\rfloor \right)\times 10^{14},1\right)\\ x_{00}=\mathrm{mod}\left(\left(Z_{3}\left(3\right)+Z_{3}\left(4\right)-\left\lfloor Z_{3}\left(3\right)+Z_{3}\left(4\right)\right\rfloor \right)\times 10^{14},1\right)\\ y_{00}=\mathrm{mod}\left(\left(Z_{3}\left(1\right)+Z_{3}\left(3\right)-\left\lfloor Z_{3}\left(1\right)+Z_{3}\left(3\right)\right\rfloor \right)\times 10^{14},1\right)\\ v_{0}=\mathrm{mod}\left(\left(Z_{3}\left(2\right)+Z_{3}\left(4\right)-\left\lfloor Z_{3}\left(2\right)+Z_{3}\left(4\right)\right\rfloor \right)\times 10^{14},1\right) \end{array}\right.$$

$\left\lfloor \cdot \right\rfloor$ represents rounding down, and $Z_{3}\left(i\right),i=1,2,3,4$ represents the *i*-th element of matrix $Z_{3}$.

#### 3.1.2. Calculating Sampling Rates Based on Multiscale Block Compressed Sensing Theory

Given the target sampling rate, the sampling rate sequences of the coefficient matrices of each layer after wavelet decomposition are calculated according to Equations (4)–(7), and denoted as subrates. In this paper, the image is decomposed by three-level wavelet transform, and there are three sampling rates.

#### 3.1.3. Generating the Measurement Matrix

In Section 3.1.1, all parameters and initial values of chaotic maps were generated, then $r_{2}$ and $y_{00}$ were brought into Equation (9), with $t+n_{1}\times n_{1}$ iterations; $r_{1}$ and $x_{00}$ were brought into Equation (8), with $t+n_{2}\times n_{2}$ iterations; $r_{0}$ and $x_{0}$ were brought into Equation (10), with $t+n_{3}\times n_{3}$ iterations. The sinusoidal value of the initial value is multiplied by a small coefficient every 2000 iterations to disturb the initial value of the next iteration, and three chaotic sequences $X_{1},X_{2},X_{3}$ are finally generated after the first t numbers are discarded. $X_{1}$, $X_{2}$, and $X_{3}$ are one-dimensional vectors of length $n_{1}\times n_{1}$, $n_{2}\times n_{2}$, and $n_{3}\times n_{3}$, respectively, where $n_{1},n_{2},n_{3}$ are determined by the block sizes given in advance. To enhance the randomness of chaotic sequences, the generated chaotic sequences are further processed as

(19) $$\left\{\begin{array}{c} X_{1}^{\prime }=1-2\times X_{1}\\ X_{2}^{\prime }=1-2\times X_{2}\\ X_{3}^{\prime }=1-2\times X_{3} \end{array}\right.$$

The generated chaotic sequences are transformed to matrix form,

(20) $$\left\{ \begin{array}{l} X_{1}^{\prime \prime }=\left(\begin{array}{ccc} X_{1}^{\prime }\left(1\right) & \ldots & X_{1}^{\prime }\left(n_{1}\right)\\ \vdots & \ddots & \vdots \\ X_{1}^{\prime }\left(n_{1}\times \left(n_{1}-1\right)+1\right) & \ldots & X_{1}^{\prime }\left(n_{1}\times n_{1}\right) \end{array}\right)\\ X_{2}^{\prime \prime }=\left(\begin{array}{ccc} X_{2}^{\prime }\left(1\right) & \ldots & X_{2}^{\prime }\left(n_{2}\right)\\ \vdots & \ddots & \vdots \\ X_{2}^{\prime }\left(n_{2}\times \left(n_{2}-1\right)+1\right) & \ldots & X_{2}^{\prime }\left(n_{2}\times n_{2}\right) \end{array}\right)\\ X_{3}^{\prime \prime }=\left(\begin{array}{ccc} X_{3}^{\prime }\left(1\right) & \ldots & X_{3}^{\prime }\left(n_{3}\right)\\ \vdots & \ddots & \vdots \\ X_{3}^{\prime }\left(n_{3}\times \left(n_{3}-1\right)+1\right) & \ldots & X_{3}^{\prime }\left(n_{3}\times n_{3}\right) \end{array}\right) \end{array}\right.$$

and the corresponding orthogonal bases $\Phi_{1},\Phi_{2},\Phi_{3}$ of $X_{1}^{\prime \prime },X_{2}^{\prime \prime },X_{3}^{\prime \prime }$, respectively, are used as redundant measurement matrices. The new row dimensions $m_{i},i=1,2,3$ are obtained by multiplying the row dimensions $n_{i},i=1,2,3$ of the redundant measurement matrices by the corresponding sampling rates. The first $m_{i}$ rows of $\Phi_{1},\Phi_{2},\Phi_{3}$ are extracted as formal measurement matrices $\Phi_{1}{}^{\prime },\Phi_{2}{}^{\prime },\Phi_{3}{}^{\prime }$.

#### 3.1.4. Encrypting the Plain Image

**Figure 2:**
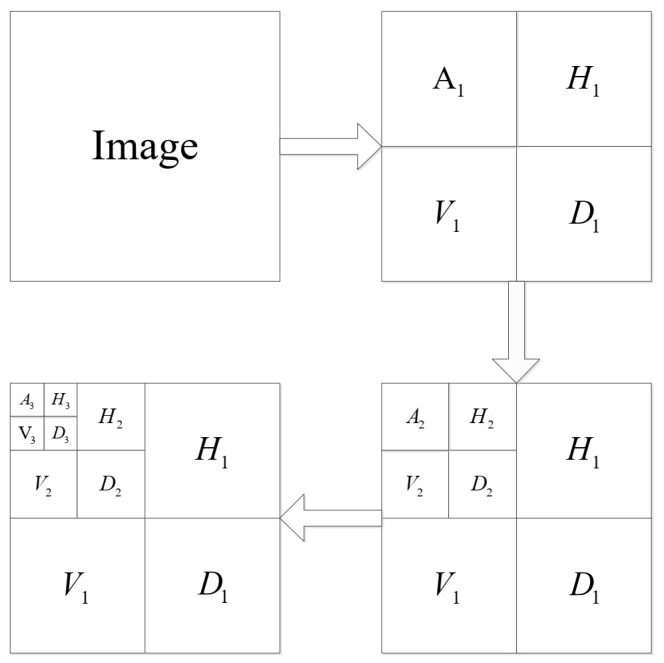
Diagram of three-level wavelet decomposition.

Step 1: After discrete wavelet decomposition of the original image, a low-frequency coefficient and nine high-frequency coefficients in horizontal, vertical, and diagonal directions are obtained. After three-layer wavelet decomposition, the ratio of the original image size $M_{0}$ to the block size of each layer is $M_{0}:M_{1}:M_{2}:M_{3}=8:4:2:1$. The three-level wavelet decomposition is shown in Figure 2.

Step 2: Given an array of length 3, the values of elements represent the block sizes. The third-, second-, and first-level wavelet decomposition coefficient matrices $H_{3},V_{3},D_{3}$, $H_{2},V_{2},D_{2}$, and $H_{1},V_{1},D_{1}$ are divided into blocks of ${\mathrm{block}\_\mathrm{size}}_{3}\times {\mathrm{block}\_\mathrm{size}}_{3}$, ${\mathrm{block}\_\mathrm{size}}_{2}\times {\mathrm{block}\_\mathrm{size}}_{2}$, and ${\mathrm{block}\_\mathrm{size}}_{1}\times {\mathrm{block}\_\mathrm{size}}_{1}$, respectively. Each block matrix is expanded to a column vector after partitioning. In this way, each block matrix in the third-, second-, and third-level wavelet decomposition coefficient matrices is transformed to a column vector of length ${\mathrm{block}\_\mathrm{size}}_{3}{}^{2}$, ${\mathrm{block}\_\mathrm{size}}_{2}{}^{2}$, and ${\mathrm{block}\_\mathrm{size}}_{1}{}^{2}$, respectively, and nine new coefficient matrices $NH_{3},NV_{3},ND_{3},NH_{2},NV_{2},ND_{2},$$NH_{1},NV_{1},ND_{1}$ are constructed by merging the corresponding column vectors.

Step 3: The three chaotic sequences $X_{1}{}^{\prime },X_{2}{}^{\prime },X_{3}{}^{\prime }$ generated in Section 3.1.3 are sorted in ascending order to obtain the corresponding index sequences ${\mathrm{Ind}}_{1},{\mathrm{Ind}}_{2},{\mathrm{Ind}}_{3}$. The coefficient matrices $A_{3},NH_{3},NV_{3},ND_{3}$, $NH_{2},NV_{2},ND_{2}$, and $NH_{1},NV_{1},ND_{1}$ of the third-, second-, and first-level wavelet decomposition, respectively, are scrambled with ${\mathrm{Ind}}_{3}$, ${\mathrm{Ind}}_{2}$, and ${\mathrm{Ind}}_{1}$, respectively, to obtain $A_{3}^{\prime },H_{3}^{\prime },V_{3}^{\prime },D_{3}^{\prime }$, $H_{2}^{\prime },V_{2}^{\prime },D_{2}^{\prime }$, and $H_{1}^{\prime },V_{1}^{\prime },D_{1}^{\prime }$. For example, if $A_{3}$ is a matrix of size m×n, we expand it to a sequence of length m×n, i is the index of the i-th element in the sequence, and

(21) $$A_{3}^{\prime }\left(Ind_{3}\left(m\times n-i+1\right)\right)=A_{3}\left(Ind_{3}\left(i\right)\right)$$

where $A_{3}^{\prime }$ is the scrambled sequence of $A_{3}$.

Step 4: The coefficient matrix $A_{3}^{\prime }$ remains unchanged. We compress the coefficient matrices $H_{3}^{\prime },V_{3}^{\prime },D_{3}^{\prime }$, $H_{2}^{\prime },V_{2}^{\prime },D_{2}^{\prime }$, and $H_{1}^{\prime },V_{1}^{\prime },D_{1}^{\prime }$ with $\Phi_{3}^{\prime }$, $\Phi_{2}^{\prime }$, and $\Phi_{1}^{\prime }$, respectively, to obtain corresponding measurement matrice s$H_{3}^{\prime \prime },V_{3}^{\prime \prime },D_{3}^{\prime \prime }$, $H_{2}^{\prime \prime },V_{2}^{\prime \prime },D_{2}^{\prime \prime }$, and $H_{1}^{\prime \prime },V_{1}^{\prime \prime },D_{1}^{\prime \prime }$, calculated by Equation (3).

Step 5: Coefficient matrices with the frequency of the three-level wavelet decomposition are combined to form the matrix to be measured,

(22) $$T=\left(\begin{array}{ccc} H_{1}^{\prime \prime } & V_{1}^{\prime \prime } & D_{1}^{\prime \prime }\\ H_{2}^{\prime \prime } & V_{2}^{\prime \prime } & D_{2}^{\prime \prime }\\ H_{3}^{\prime \prime } & V_{3}^{\prime \prime } & D_{3}^{\prime \prime } \end{array}\right)$$

To enhance the randomness, T is processed according to the information $l_{0},l_{1},L_{0},L_{1}$ obtained in Section 3.1.1 to obtain

(23) $$\left\{\begin{array}{l} NT=-T,l_{0}>l_{1}\\ NT=T-0.5,L_{0}>L_{1}\\ NT=T+0.5,L_{0}<L_{1} \end{array}\right.$$

Step 6: According to the method proposed in Section 2.3.2, RSTPM and CSTPM are constructed according to the element information of matrix NT.

Step 7: The maximum and minimum values of nine coefficient matrices,$H_{i}^{\prime \prime },V_{i}^{\prime \prime },D_{i}^{\prime \prime },i=1,2,3$, after wavelet decomposition, are obtained. The coefficient matrices are quantized to the interval [0,255],

(24) $$M^{\prime }=floor\left(\frac{255\times \;\left(M-\min\right)}{\left(\max-\min\right)}\right)$$

where min and max are the minimum and maximum values, respectively, of M. The coefficient matrices $H_{i}^{\prime \prime },V_{i}^{\prime \prime },D_{i}^{\prime \prime },i=1,2,3$ are quantized to obtain corresponding matrices $H_{i}^{\prime \prime \prime },V_{i}^{\prime \prime \prime },D_{i}^{\prime \prime \prime },i=1,2,3$. The low-frequency coefficient matrix $A_{3}^{\prime }$ is decomposed by SVD to obtain three sub-matrices u,s,v of the same dimension, whose maximum and minimum values are obtained. The sub-matrices are quantized to the interval [0,255] by Equation (24) to obtain corresponding matrices U, S, VT.

Step 8: We combine the high frequency coefficients of each layer into groups in the same direction: $H_{1}^{\prime \prime \prime },H_{2}^{\prime \prime \prime },H_{3}^{\prime \prime \prime }$ are in the first group, $V_{1}^{\prime \prime \prime },V_{2}^{\prime \prime \prime },V_{3}^{\prime \prime \prime }$ are in the second group, and $D_{1}^{\prime \prime \prime },D_{2}^{\prime \prime \prime },D_{3}^{\prime \prime \prime }$ are in the third group. The index values are obtained using the chaotic sequences $X_{1}^{\prime },X_{2}^{\prime },X_{3}^{\prime }$ generated in Section 3.1.3,

(25) $$\left\{ \begin{array}{l} Lind_{1}=\left(X_{1}^{\prime }\left(n_{1}\times n_{1}\right)+X_{2}^{\prime }\left(n_{2}\times n_{2}\right)+X_{3}^{\prime }\left(n_{3}\times n_{3}\right)\right)/3\\ {Lind}_{1}^{\prime }=floor\left(\mathrm{mod}\left(abs\left(Lind_{1}\right)\times 10^{8},6\right)\right)+1\\ Lind_{2}=\left(X_{1}^{\prime }\left(n_{1}\times n_{1}-1\right)+X_{2}^{\prime }\left(n_{2}\times n_{2}-1\right)+X_{3}^{\prime }\left(n_{3}\times n_{3}-1\right)\right)/3\\ {Lind}_{2}^{\prime }=floor\left(\mathrm{mod}\left(abs\left(Lind_{2}\right)\times 10^{8},6\right)\right)+1\\ Lind_{3}=\left(X_{1}^{\prime }\left(n_{1}\times n_{1}-2\right)+X_{2}^{\prime }\left(n_{2}\times n_{2}-2\right)+X_{3}^{\prime }\left(n_{3}\times n_{3}-2\right)\right)/3\\ {Lind}_{3}^{\prime }=floor\left(\mathrm{mod}\left(abs\left(Lind_{3}\right)\times 10^{8},6\right)\right)+1 \end{array}\right.$$

That is, the index values ${Lind}_{1}^{\prime }$, ${Lind}_{2}^{\prime }$, and ${Lind}_{3}^{\prime }$ of the first through third groups, respectively, are determined by the last, penultimate, and antepenultimate elements of $X_{1}^{\prime },X_{2}^{\prime },X_{3}^{\prime }$. The three index values are mapped to the interval [1,6], which indicates that there are six possible permutations for each group,

(26) $$\left\{ \begin{array}{l} \left(\begin{array}{l} Y_{11}\\ Y_{12}\\ Y_{13} \end{array}\right)=\left(\begin{array}{l} H_{3}^{\prime \prime \prime }\\ H_{2}^{\prime \prime \prime }\\ H_{1}^{\prime \prime \prime } \end{array}\right),\left(\begin{array}{l} Y_{21}\\ Y_{22}\\ Y_{23} \end{array}\right)=\left(\begin{array}{l} V_{3}^{\prime \prime \prime }\\ V_{2}^{\prime \prime \prime }\\ V_{1}^{\prime \prime \prime } \end{array}\right),\left(\begin{array}{l} Y_{{}_{31}}\\ Y_{32}\\ Y_{33} \end{array}\right)=\left(\begin{array}{l} D_{3}^{\prime \prime \prime }\\ D_{2}^{\prime \prime \prime }\\ D_{1}^{\prime \prime \prime } \end{array}\right)\\ \left(\begin{array}{l} Y_{11}\\ Y_{12}\\ Y_{13} \end{array}\right)=\left(\begin{array}{l} H_{3}^{\prime \prime \prime }\\ H_{1}^{\prime \prime \prime }\\ H_{2}^{\prime \prime \prime } \end{array}\right),\left(\begin{array}{l} Y_{21}\\ Y_{22}\\ Y_{23} \end{array}\right)=\left(\begin{array}{l} V_{3}^{\prime \prime \prime }\\ V_{1}^{\prime \prime \prime }\\ V_{2}^{\prime \prime \prime } \end{array}\right),\left(\begin{array}{l} Y_{{}_{31}}\\ Y_{32}\\ Y_{33} \end{array}\right)=\left(\begin{array}{l} D_{3}^{\prime \prime \prime }\\ D_{1}^{\prime \prime \prime }\\ D_{2}^{\prime \prime \prime } \end{array}\right)\\ \left(\begin{array}{l} Y_{11}\\ Y_{12}\\ Y_{13} \end{array}\right)=\left(\begin{array}{l} H_{2}^{\prime \prime \prime }\\ H_{3}^{\prime \prime \prime }\\ H_{1}^{\prime \prime \prime } \end{array}\right),\left(\begin{array}{l} Y_{21}\\ Y_{22}\\ Y_{23} \end{array}\right)=\left(\begin{array}{l} V_{2}^{\prime \prime \prime }\\ V_{3}^{\prime \prime \prime }\\ V_{1}^{\prime \prime \prime } \end{array}\right),\left(\begin{array}{l} Y_{{}_{31}}\\ Y_{32}\\ Y_{33} \end{array}\right)=\left(\begin{array}{l} D_{2}^{\prime \prime \prime }\\ D_{3}^{\prime \prime \prime }\\ D_{1}^{\prime \prime \prime } \end{array}\right)\\ \left(\begin{array}{l} Y_{11}\\ Y_{12}\\ Y_{13} \end{array}\right)=\left(\begin{array}{l} H_{2}^{\prime \prime \prime }\\ H_{1}^{\prime \prime \prime }\\ H_{3}^{\prime \prime \prime } \end{array}\right),\left(\begin{array}{l} Y_{21}\\ Y_{22}\\ Y_{23} \end{array}\right)=\left(\begin{array}{l} V_{2}^{\prime \prime \prime }\\ V_{1}^{\prime \prime \prime }\\ V_{3}^{\prime \prime \prime } \end{array}\right),\left(\begin{array}{l} Y_{{}_{31}}\\ Y_{32}\\ Y_{33} \end{array}\right)=\left(\begin{array}{l} D_{2}^{\prime \prime \prime }\\ D_{1}^{\prime \prime \prime }\\ D_{3}^{\prime \prime \prime } \end{array}\right)\\ \left(\begin{array}{l} Y_{11}\\ Y_{12}\\ Y_{13} \end{array}\right)=\left(\begin{array}{l} H_{1}^{\prime \prime \prime }\\ H_{3}^{\prime \prime \prime }\\ H_{2}^{\prime \prime \prime } \end{array}\right),\left(\begin{array}{l} Y_{21}\\ Y_{22}\\ Y_{23} \end{array}\right)=\left(\begin{array}{l} V_{1}^{\prime \prime \prime }\\ V_{3}^{\prime \prime \prime }\\ V_{2}^{\prime \prime \prime } \end{array}\right),\left(\begin{array}{l} Y_{{}_{31}}\\ Y_{32}\\ Y_{33} \end{array}\right)=\left(\begin{array}{l} D_{1}^{\prime \prime \prime }\\ D_{3}^{\prime \prime \prime }\\ D_{2}^{\prime \prime \prime } \end{array}\right)\\ \left(\begin{array}{l} Y_{11}\\ Y_{12}\\ Y_{13} \end{array}\right)=\left(\begin{array}{l} H_{1}^{\prime \prime \prime }\\ H_{2}^{\prime \prime \prime }\\ H_{3}^{\prime \prime \prime } \end{array}\right),\left(\begin{array}{l} Y_{21}\\ Y_{22}\\ Y_{23} \end{array}\right)=\left(\begin{array}{l} V_{1}^{\prime \prime \prime }\\ V_{2}^{\prime \prime \prime }\\ V_{3}^{\prime \prime \prime } \end{array}\right),\left(\begin{array}{l} Y_{{}_{31}}\\ Y_{32}\\ Y_{33} \end{array}\right)=\left(\begin{array}{l} D_{1}^{\prime \prime \prime }\\ D_{2}^{\prime \prime \prime }\\ D_{3}^{\prime \prime \prime } \end{array}\right) \end{array}\right.$$

The first through third groups after sorting are being recorded as $Y_{1}$, $Y_{2}$, and $Y_{3}$, respectively, whose internal three block matrices are recorded as $Y_{11},Y_{12},Y_{13}$, $Y_{21},Y_{22},Y_{23}$, and $Y_{31},Y_{32},Y_{33}$.

Step 9: The quantization matrices U, S, and VT obtained from Step 7 are inserted in the first through third groups of matrices, respectively, after the first round of combination. The index values are obtained using the three chaotic sequences $X_{1}^{\prime },X_{2}^{\prime },X_{3}^{\prime }$ generated in Section 3.1.3,

(27) $$\left\{\begin{array}{l} Hind_{1}=\;\left(X_{1}^{\prime }\left(1\right)+X_{2}^{\prime }\left(1\right)+X_{3}^{\prime }\left(1\right)\right)/3\\ Hind_{2}=\;\left(X_{1}^{\prime }\left(2\right)+X_{2}^{\prime }\left(2\right)+X_{3}^{\prime }\left(2\right)\right)/3\\ Hind_{3}=\;\left(X_{1}^{\prime }\left(3\right)+X_{2}^{\prime }\left(3\right)+X_{3}^{\prime }\left(3\right)\right)/3\\ {Hind}_{1}^{\prime }=floor\left(\mathrm{mod}\left(abs\left(Hind_{1}\right)\times 10^{8},4\right)\right)+1\\ {Hind}_{2}^{\prime }=floor\left(\mathrm{mod}\left(abs\left(Hind_{2}\right)\times 10^{8},4\right)\right)+1\\ {Hind}_{3}^{\prime }=floor\left(\mathrm{mod}\left(abs\left(Hind_{3}\right)\times 10^{8},4\right)\right)+1 \end{array}\right.$$

That is, the index values ${Hind}_{1}^{\prime }$, ${Hind}_{2}^{\prime }$, and ${Hind}_{3}^{\prime }$ of the first through third groups, respectively, are determined by the first through third elements, respectively, of $X_{1}^{\prime },X_{2}^{\prime },X_{3}^{\prime }$. At the same time, the three index values are mapped to the interval [1,4], which indicates that there are four possible permutations for each group: the top, the gaps between two adjacent block matrices, and the bottom,

(28) $$\left\{ \begin{array}{l} Y_{1}^{\prime }=\left(\begin{array}{l} U\\ Y_{11}\\ Y_{12}\\ Y_{13} \end{array}\right),Y_{2}^{\prime }=\left(\begin{array}{l} S\\ Y_{21}\\ Y_{22}\\ Y_{23} \end{array}\right),Y_{3}^{\prime }=\left(\begin{array}{l} VT\\ Y_{31}\\ Y_{32}\\ Y_{33} \end{array}\right)\\ Y_{1}^{\prime }=\left(\begin{array}{l} Y_{11}\\ U\\ Y_{12}\\ Y_{13} \end{array}\right),Y_{2}^{\prime }=\left(\begin{array}{l} Y_{21}\\ S\\ Y_{22}\\ Y_{23} \end{array}\right),Y_{3}^{\prime }=\left(\begin{array}{l} Y_{31}\\ VT\\ Y_{32}\\ Y_{33} \end{array}\right)\\ Y_{1}^{\prime }=\left(\begin{array}{l} Y_{11}\\ Y_{12}\\ U\\ Y_{13} \end{array}\right),Y_{2}^{\prime }=\left(\begin{array}{l} Y_{21}\\ Y_{22}\\ S\\ Y_{23} \end{array}\right),Y_{3}^{\prime }=\left(\begin{array}{l} Y_{31}\\ Y_{32}\\ VT\\ Y_{33} \end{array}\right)\\ Y_{1}^{\prime }=\left(\begin{array}{l} Y_{11}\\ Y_{12}\\ Y_{13}\\ U \end{array}\right),Y_{2}^{\prime }=\left(\begin{array}{l} Y_{21}\\ Y_{22}\\ Y_{23}\\ S \end{array}\right),Y_{3}^{\prime }=\left(\begin{array}{l} Y_{31}\\ Y_{32}\\ Y_{33}\\ VT \end{array}\right) \end{array}\right.$$

We mark the first through third group matrices after combination as $Y_{1}^{\prime }$, $Y_{2}^{\prime }$, and $Y_{3}^{\prime }$, respectively, and combine them as

(29) $$T^{\prime }=\left(\begin{array}{ccc} Y_{1}^{\prime } & Y_{2}^{\prime } & Y_{3}^{\prime } \end{array}\right)$$

Step 10: The dimension of the matrix should be adjusted to prevent attackers from obtaining the information of the encryption scheme through the dimension of cipher images. Suppose the dimension of the merged matrix is m×n. We find two factors $m_{1}$ and $n_{1}$ of m×n such that $m_{1}\times n_{1}=m\times n$, minimize $\left|m_{1}-n_{1}\right|$, and adjust the dimension of the matrix to $m_{1}\times n_{1}$, obtaining the matrix information

(30) $$\left\{\begin{array}{l} \max=\max\left(T^{\prime }\right)\\ \min=\min\left(T^{\prime }\right)\\ d_{1}=\;\mathrm{floor}\left(\mathrm{mean}\left(T^{\prime }\right)\right)\\ d_{2}=\;\mathrm{ceil}\left(\left(\max+\min\right)/2\right)\\ d_{1}{}^{\prime }=\mathrm{mod}\left(d_{1},10\right)\\ d_{2}{}^{\prime }=\mathrm{mod}\left(d_{2},10\right)\\ d_{12}=\max\left(d_{1}{}^{\prime },d_{2}{}^{\prime }\right) \end{array}\right.$$

Step 11: The parameters a, b, c and initial value $v_{0}$ generated in Section 3.1.1 are brought into Equation (11), iterating $t+\;\left(d_{12}+1\right)\times \;m_{1}\times n_{1}$ times, and the sinusoidal value of the initial state is multiplied by a small coefficient every 2000 iterations to disturb the initial value of the next iteration. The chaotic sequence V is generated after the first t numbers are discarded. To enhance the randomness, we determine the chaotic sequence

(31) $$V^{\prime }=1-4\times V$$

according to which we generate sub-sequences $V_{1},V_{2},V_{3},V_{4}$,

(32) $$\left\{\begin{array}{l} V_{1}=10\times V^{\prime }-round\left(10\times V^{\prime }\right)\\ V_{2}=10^{2}\times V^{\prime }-round\left(10^{2}\times V^{\prime }\right)\\ V_{3}=10^{3}\times V^{\prime }-round\left(10^{3}\times V^{\prime }\right)\\ V_{4}=10^{4}\times V^{\prime }-round\left(10^{4}\times V^{\prime }\right) \end{array}\right.$$

The control parameters of the scrambling process are generated according to $V_{1},V_{2},V_{3},V_{4}$ as

(33) w1=fixmodV1m1′+n1′+1,5w2=fixmodV2roundm1′2+n1′2+2×102,52w3=fixmodV3absroundm1′3−n1′3+3×103,53w4=fixmodV4absroundm1′4−n1′4+4×104,54

where $w_{1}∼w_{4}$ are used for subsequent shift operations, fix is a function that rounds toward zero, and $m_{1}^{\prime }$ and $n_{1}^{\prime }$ are the largest prime factors of $m_{1}$ and $n_{1}$, respectively.

Step 12: The scrambling operations are based on RSTPM, as generated in Step 6. The shift numbers are $w_{1}$–$w_{4}$ when column coordinates are the po-od, po-ev, ne-od, and ne-ev numbers, respectively. The scrambling operations are shown in Table 4. The up arrow means move up, the down arrow means move down. We record the scrambled matrix as $T^{\prime \prime }$ and set the initial transition flag bit matrix and all element values to zero. If the element of a position is shifted, then the flag bits of the corresponding position change from 0 to 1. Taking the state transition probability matrix generated in Table 3 as an example, the positions with probability values greater than 0.25 are in the ne-od number and po-ev number columns. That is, the probability values of the two middle columns are selected. Therefore, the flag bits of these two columns are set to 1.

Step 13: The scrambling operations are based on CSTPM, as generated in Step 6. The shift numbers are $w_{1}$, $w_{3}$, $w_{2}$, and $w_{4}$, if the column coordinates are the po-od, ne-od, po-ev, and ne-ev numbers, respectively. We record the scrambled matrix as $T^{\prime \prime \prime }$. We set the state transition flag bits in the same way. If the element of a position is shifted, then the flag bits of the corresponding position change from 0 to 1.

Step 14: The chaotic sequence $V^{\prime }$ generated in Step 11 is quantized to the interval [0,255] to get the new matrix $V^{\prime \prime }$. The matrix information $d_{1}^{\prime }$ and $d_{2}^{\prime }$ obtained in Step 10 is used to generate the chaotic sequences, $V^{\prime \prime }$ is sampled at an interval of $d_{1}^{\prime }$ to obtain sequence $V_{1}^{\prime }$, and $V^{\prime \prime }$ is sampled at an interval of $d_{2}^{\prime }$ to obtain sequence $V_{1}^{\prime }$,

(34) $$\left\{\begin{array}{c} V_{1}^{\prime }\left(i+1\right)=V^{\prime \prime }\left(i\times d_{1}^{\prime }+1\right)\\ V_{2}^{\prime }\left(i+1\right)=V^{\prime \prime }\left(i\times d_{2}^{\prime }+1\right) \end{array}\right.$$

We take the position of the last element of sequence $V_{2}^{\prime }$ as the start position, and take the consecutive $m_{1}\times n_{1}$ elements from sequence $V^{\prime \prime }$ and record them as sequence $V_{0}$,

(35) $$V_{0}=V^{\prime \prime }\left(\left(\left(m_{1}\times n_{1}-1\right)\times d_{2}^{\prime }+2\right):\left(\left(m_{1}\times n_{1}-1\right)\times \left(d_{2}^{\prime }+1\right)+2\right)\right)$$

We perform an XOR operation between matrix $T^{\prime \prime \prime }$ and sequence $V_{0}$,

(36) $${T^{\prime \prime \prime }}^{\prime }\left(i\right)\;=T^{\prime \prime \prime }\left(i\right)⊕V_{0}\left(m_{1}\times n_{1}-i+1\right)$$

and denote ${T^{\prime \prime \prime }}^{\prime }$ as A.

Step 15: The matrix A is changed with front addition and a modular operation to obtain the matrix B, using the sequence $V_{1}^{\prime }$ generated in Step 14. We perform a cyclic left shift on B to obtain $B^{\prime }$, i.e., and the definition of BitCircShift is shown in Algorithm 1.

(37) $$\left\{\begin{array}{l} B\left(i\right)\;=\mathrm{mod}\left(B\left(i-1\right)+V_{1}^{\prime }\left(i\right)+A\left(i\right),256\right)\\ B^{\prime }\left(i\right)=BitCircShift\left(B\left(i\right),\mathrm{mod}\left(B\left(i-1\right),8\right)\right) \end{array}\right.$$

$B^{\prime }$ is changed through back addition and a modular operation to get *C*, using the sequence $V_{2}^{\prime }$ generated in Step 14. We perform a cyclic left shift on *C* to obtain $C^{\prime }$, i.e.,

(38) $$\left\{\begin{array}{l} C\left(i\right)\;=\mathrm{mod}\left(C\left(i+1\right)+V_{2}^{\prime }\left(i\right)+B^{\prime }\left(i\right),256\right)\\ C^{\prime }\left(i\right)=BitCircShift\left(C\left(i\right),\mathrm{mod}\left(C\left(i+1\right),8\right)\right) \end{array}\right.$$

At this point, the final cipher image is obtained.
**Algorithm 1.** The BitCircShift Operation**Input:** The number to be shifted **x** and the shift number **k**. **Output:** The number after shift **y**.1: **if** abs(**k**)>7 || **k**==0 **then** 2:   **y**←**x** 3: **end if** 4: **if k**>0 **then** 5: y1←$2^{k}x$ mod 256 6:   y2← floor($x/2^{8-k}$) 7: **else** 8:   y1← floor($2^{k}x$) 6:   y2←(**x** mod $2^{-k}$)×$2^{8+k}$ 9: **end if** 10: **y**←y1 + y2 11: **end**

### 3.2. Decryption Process

**Figure 3:**
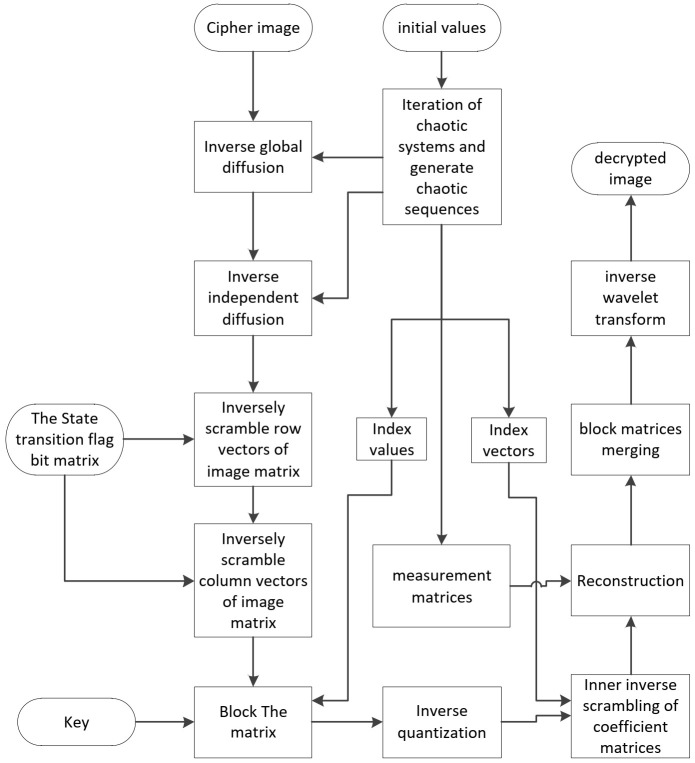
Flowchart of image decryption process.

The image decryption scheme is the reverse of image encryption. The process is controlled by the key, including the initial values of chaotic systems, state transition flag bit matrix, and maximum and minimum of matrices, as received by the sender. The process is shown in Figure 3.

Step 1: The cipher image matrix is expanded to a sequence *C*, and then we perform a cyclic right shift operation on *C* to obtain *D*, which is changed with inverse back addition and a modular operation to get the sequence $D^{\prime }$, using the sequence $V_{2}{}^{\prime }$ generated before,

(39) $$\left\{\begin{array}{l} D\left(i\right)\;=BitCircShift\left(C\left(i\right),-\mathrm{mod}\left(C\left(i+1\right),8\right)\right)\\ D^{\prime }\left(i\right)\;=\mathrm{mod}\left(256\times 2+D\left(i\right)-C\left(i+1\right)-V_{2}^{\prime }\left(i\right),256\right) \end{array}\right.$$

We perform a cyclic right shift operation on $D^{\prime }$ to obtain E, which is changed with inverse front addition and a modular operation to get the sequence $E^{\prime }$ by using the sequence $V_{1}^{\prime }$ generated before,

(40) $$\left\{\begin{array}{l} E\left(i\right)\;=BitCircShift\left(D^{\prime }\left(i\right),-\mathrm{mod}\left(D^{\prime }\left(i-1\right),8\right)\right)\\ E^{\prime }\left(i\right)\;=\mathrm{mod}\left(256\times 2+E\left(i\right)-D^{\prime }\left(i-1\right)-V_{1}^{\prime }\left(i\right),256\right) \end{array}\right.$$

Step 2: We perform an XOR operation between sequences $E^{\prime }$ and $V_{0}$ to obtain sequence $E^{\prime \prime }$,

(41) $$E^{\prime \prime }\left(i\right)\;=E^{\prime }\left(i\right)⊕V_{0}\left(m_{1}\times n_{1}-i+1\right)$$

and transform $E^{\prime \prime }$ to a matrix of size $m_{1}\times n_{1}$.

Step 3: $E^{\prime \prime }$ is inversely scrambled according to the previously generated transition flag bit matrix, and we find the positions of its elements whose values are 1. We inversely scramble the matrix according to the rule described in Section 2.3.2. The matrix is recorded as $E^{\prime \prime \prime }$ after inversely scrambling row vectors, and that matrix’s column vectors are inversely scrambled to obtain ${E^{\prime \prime \prime }}^{\prime }$.

Step 4: Find the positions of the U, S, VT and coefficient matrices $H_{i}^{\prime \prime },V_{i}^{\prime \prime },D_{i}^{\prime \prime },i=1,2,3$ in matrix ${E^{\prime \prime \prime }}^{\prime }$ according to ${\mathrm{Hind}}_{1}^{\prime }∼{\mathrm{Hind}}_{3}^{\prime }$ and ${\mathrm{Lind}}_{1}^{\prime }∼{\mathrm{Lind}}_{3}^{\prime }$, then we divide the matrix ${E^{\prime \prime \prime }}^{\prime }$ into blocks to obtain them. The sub-matrices of low-frequency coefficient matrices U,S,VT are inversely quantized to obtain corresponding matrices $U^{\prime },S^{\prime },VT^{\prime }$, and the coefficient matrices ${HH}_{i}^{\prime },{VV}_{i}^{\prime },{DD}_{i}^{\prime },i=1,2,3$ are obtained by inverse quantization of corresponding matrices $H_{i}^{\prime \prime },V_{i}^{\prime \prime },D_{i}^{\prime \prime },i=1,2,3$, where quantization is expressed as

(42) $$M=M^{\prime }\times \left(\max-\min\right)/255+\min$$

where max and min are the maximum and minimum values, respectively, of M. The low-frequency coefficient matrix $A_{3}^{\prime }$ is obtained by calculating the product of the three sub-matrices,

(43) $$A_{3}^{\prime }=U^{\prime }\times S’\times VT^{\prime }$$

Step 5: We first generate index vectors ${\mathrm{Ind}}_{1}∼{\mathrm{Ind}}_{3}$. Coefficient matrices $A_{3}^{\prime },{HH}_{3}^{\prime },$${VV}_{3}^{\prime },{DD}_{3}^{\prime }$, ${HH}_{2}^{\prime },{VV}_{2}^{\prime },{DD}_{2}^{\prime }$, and ${HH}_{1}^{\prime },{VV}_{1}^{\prime },{DD}_{1}^{\prime }$ are inversely scrambled with ${\mathrm{Ind}}_{3}$, ${\mathrm{Ind}}_{2}$, and ${\mathrm{Ind}}_{1}$, respectively. For example, we expand $A_{3}^{\prime }$ of size m×n to a sequence of length m×n, whose *i*-th element is

(44) $$A_{3}\left(Ind_{3}\left(i\right)\right)\;=A_{3}^{\prime }\left(Ind_{3}\left(m\times n-i+1\right)\right)$$

where $A_{3}$ is the scrambled sequence of $A_{3}{}^{\prime }$.

Step 6: We reconstruct the image with the SPL algorithm. The column vectors of the reconstructed coefficient matrices of each layer are restored to block matrices according to the corresponding block size. Block matrices of size ${\mathrm{block}\_\mathrm{size}}_{3}\times {\mathrm{block}\_\mathrm{size}}_{3}$, ${\mathrm{block}\_\mathrm{size}}_{2}\times {\mathrm{block}\_\mathrm{size}}_{2}$, and ${\mathrm{block}\_\mathrm{size}}_{1}\times {\mathrm{block}\_\mathrm{size}}_{1}$ are, respectively, combined into third-, second-, and first-level wavelet decomposition coefficient matrices $A_{3},H_{3},V_{3},D_{3}$, $H_{2},V_{2},D_{2}$, and $H_{1},V_{1},D_{1}$, which are combined into a new matrix according to Figure 2.

Step 7: The matrix after inverse wavelet transform is the final decrypted image.

## 4. Simulation Results

### 4.1. Encryption and Decryption Results

**Figure 4:**
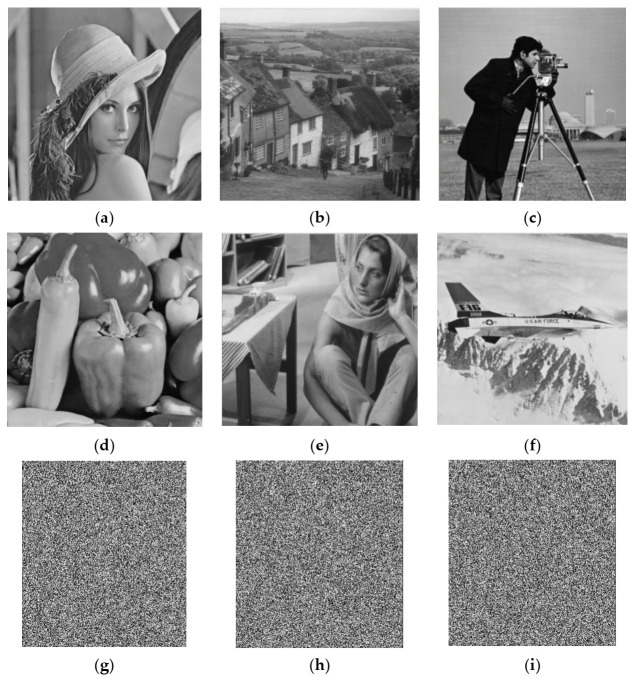

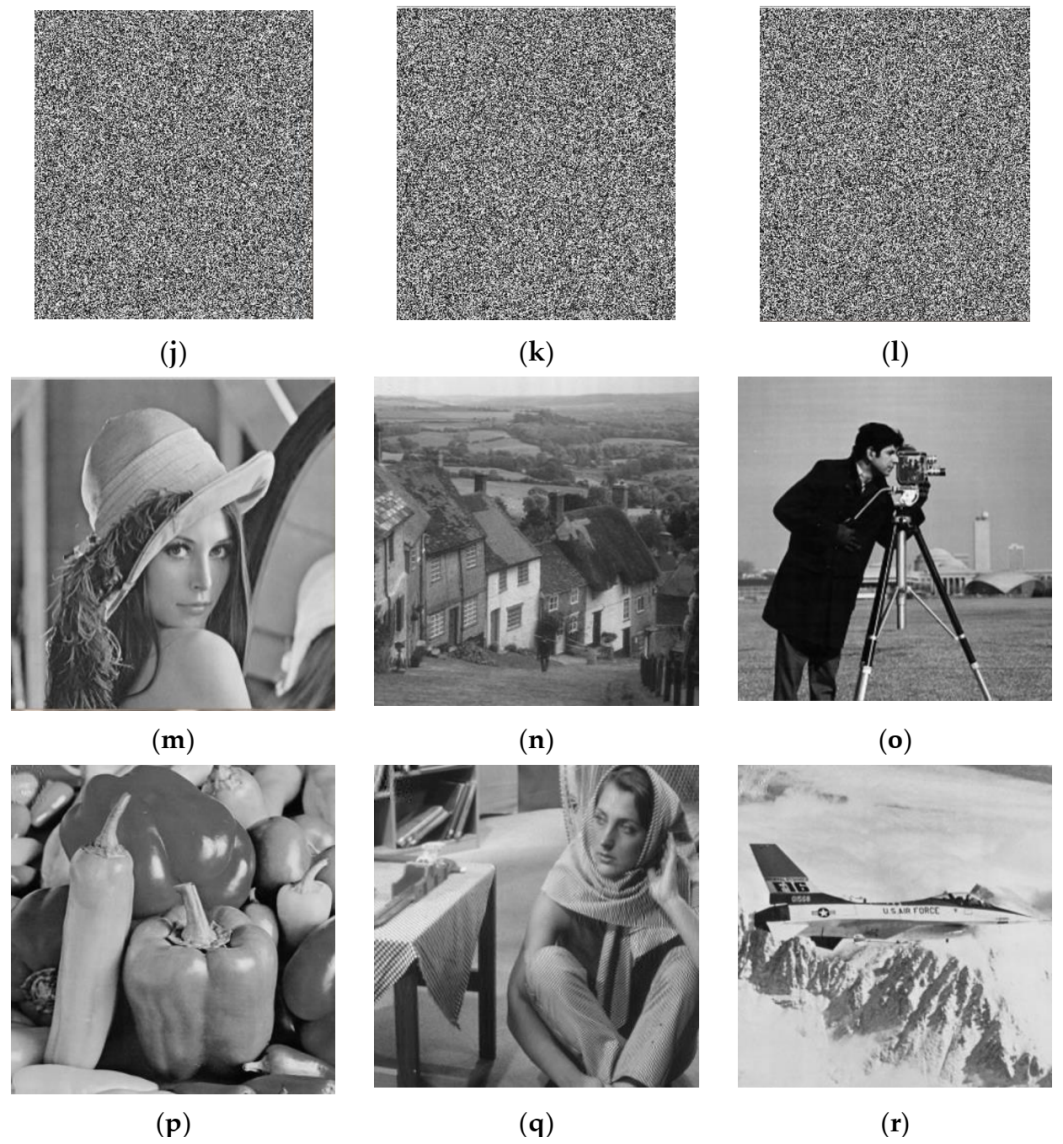
Experimental results: (**a**–**f**) plain images Lena, Goldhill, Cameraman, Peppers, Barbara, Jet; (**g**–**l**) corresponding cipher images; (**m**–**r**) corresponding decrypted images.

Simulation experiments were carried out on a laptop computer with an Intel Core i5-6200U CPU at 2.3 GHz and 4 GB RAM, on the MATLAB R2015a platform. Images of size 512 × 512, including Lena, Goldhill, Cameraman, Peppers, Barbara, and Jet, were selected as test images. The external keys were $t_{1}=0.11,t_{2}=0.22,t_{3}=0.33,$$t_{4}=0.44,t_{5}=2.723,t_{6}=0.618,t_{7}=3.141,t_{8}=4.6692,t\;=600$. The array block_size, which determines the block sizes of the image matrix, was set to [8,16,32], i.e.,${\mathrm{block}\_\mathrm{size}}_{1}=32,{\mathrm{block}\_\mathrm{size}}_{2}=16,{\mathrm{block}\_\mathrm{size}}_{3}=8$. The target sampling rate was 0.25. The experimental results of plain, cipher, and decrypted images are shown in Figure 4. The cipher images were meaningless and unrecognizable noise-like images with little connection to the plain images. Valid information about the original images cannot be obtained from the corresponding cipher images. The encryption scheme adjusted the dimensions of cipher images to 288 × 256, which further hid the information of the plain image and the encryption method. The decrypted images were meaningful images that could be clearly identified and were quite similar to the original images. Hence, the proposed scheme had good encryption and decryption effects.

### 4.2. PSNR between Plain and Decrypted Images under Different Sampling Rates

The peak signal to noise ratio (PSNR) is used to objectively judge the quality of a decrypted image. A larger PSNR indicates a smaller difference between plain and decrypted images, and higher reconstruction accuracy. For gray images,

(45) $$\left\{\begin{array}{l} \mathrm{PSNR}\;=10\times \log_{10}\frac{255\times 255}{\sqrt{\mathrm{MSE}}}\\ \mathrm{MSE}\;=\frac{1}{M\times N}\sum_{i=1}^{M}\sum_{j=1}^{N}{\left[X\left(i,j\right)-Y\left(i,j\right)\right]}^{2} \end{array}\right.$$

where M and N are the row dimension and column dimension of the image, and $X\left(i,j\right)$ and $Y\left(i,j\right)$ are the pixel values of the plain image X and decrypted image Y, respectively, at position $\left(i,j\right)$.

In this experiment, 512 × 512 images, including Lena, Cameraman, Peppers, and Couple, were selected as test images, and PSNR values between plain and decrypted images were measured at different sampling rates. Table 5 compares partial experimental results with those in Gan et al. [10]. The scheme of Gan et al. [10] adopts traditional compressed sensing theory, using one sampling rate for the whole image, which reveals that the distribution differences of image information between low- and high-frequency coefficients are not fully considered, and the resulting decrypted image does not have high reconstruction quality. The image reconstruction effect of the proposed scheme is better than that of the scheme in Gan et al. [10]. Table 6 compares some experimental results with those in Luo et al. [28]. In the latter, the plain image is first decomposed into approximate and detail components by discrete wavelet transform (DWT). All pixels in the approximate component are retained, and the remaining detailed components are measured by measurement matrices. A lower sampling rate is adapted to the horizontal direction decomposition coefficient (LH) and diagonal direction decomposition coefficient (HH), and a larger sampling rate is adapted to the vertical direction decomposition coefficient (HL). The reconstruction quality of the decrypted image is improved. However, there are some limitations because the sampling rates are set artificially. From Table 6, it can be seen that the proposed algorithm can better improve the reconstruction quality of the decrypted images.

### 4.3. Influence of Wavelet Basis on Image Reconstruction Effect (PSNR)

In this experiment, 12 images were decomposed by DWT in different wavelet bases, including the commonly used Symlets8, Haar, and CDF9/7. The PSNR values of the decrypted images were measured, with results as shown in Table 7. The experimental results show that the CDF9/7 wavelet base performs better in most cases; hence, this was chosen as the wavelet base.

### 4.4. Time Complexity Analysis

An algorithm should have fast encryption and decryption speeds to meet real-time needs. In this experiment, the plain image Lena with size of 512 × 512 was used as a test image, the sampling rate was set to 0.25, and the running time of each process of the scheme was measured. The results are shown in Table 8. In addition, the running times of the whole encryption process and decryption process at different sampling rates were also measured and the results are shown in Table 9. We can learn from the experimental result that the processes of iteration of chaotic systems and image reconstruction consume most of the time in the algorithm. Additionally, as the sampling rate increases, the encryption time also increases, but the decryption time is basically the same. More concretely, in encryption algorithm illustrated in Section 3.1, iteration of chaos includes Section 3.1.3 and Step 11 in Section 3.1.4, the compression process is Step 4 in Section 3.1.4, the first round of permutation is Step 3 in Section 3.1.4, the second round of permutation is Step 12 and Step 13 in Section 3.1.4, and the diffusion includes Step 14 and Step 15. The decryption process illustrated in Section 3.2 is the inverse process of the encryption process, and the compression process is replaced by the reconstruction process.

In what follows, we analyzed the time complexity of our encryption algorithm in Section 3.1 in detail. Assume the size of plain image is m×n, the block sizes of the third-, second-, and first-level wavelet decomposition coefficient matrices are $n_{1},n_{2},n_{3}$, respectively, and the target sampling rate is CR. Section 3.1.3 is to generate three chaotic sequences for scrambling, and time complexity is $\Theta \left(n_{1}{}^{4}+n_{2}{}^{4}+n_{3}{}^{4}\right)$.Step 11 in Section 3.1.4 is to generate the chaotic sequence for diffusion, and time complexity is $\Theta \left(\mathrm{CR}\times m\times n\right)$. Step 3 in Section 3.1.4 is to scramble the coefficient matrices with index sequences, and time complexity is $\Theta \left(m\times n\right)$. Step 6 in Section 3.1.4 is to generate the RSTPM and CSTPM, and time complexity is $\Theta \left(2\times \mathrm{CR}\times m\times n\right)$. Step 12 and Step 13 in Section 3.1.4 are to scramble the matrix of measurement values after CS with RSTPM and CSTPM, and time complexity is $\Theta \left(\mathrm{CR}\times m\times n\right)$. Step 14 and Step 15 in Section 3.1.4 are to diffuse the matrix after scrambling, and time complexity is $\Theta \left(3\times \mathrm{CR}\times m\times n\right)$. For the computational cost of the proposed method determined in other steps, the time complexity is about $\Theta \left(3\times \mathrm{CR}\times m\times n\right)$. From the result shown in Table 8, the total time complexity is approximately equal to $\Theta \left(5\times m\times n\right)$. Comparing with the encryption algorithms in [49,50] listed in Table 10, our scheme has a smaller time complexity. However, the time complexity of our scheme is equal to that of [10].

## 5. Security Analysis

### 5.1. Key Space

To withstand a brute-force attack, an encryption scheme should have a large key space. If the calculation accuracy of the computer is $10^{-14}$, the external key is $t_{1}∼t_{8}$, accounted for ${\left(10^{14}\right)}^{8}=10^{112}$ key space. Table 11 compares the proposed scheme and other schemes. Since the ideal key space is suggested to be at least $2^{100}<10^{31}$ for a good cryptosystem [9], the result illustrates that the key space of the proposed scheme is large enough to resist all kinds of attacks.

### 5.2. Histogram Analysis

**Figure 5:**
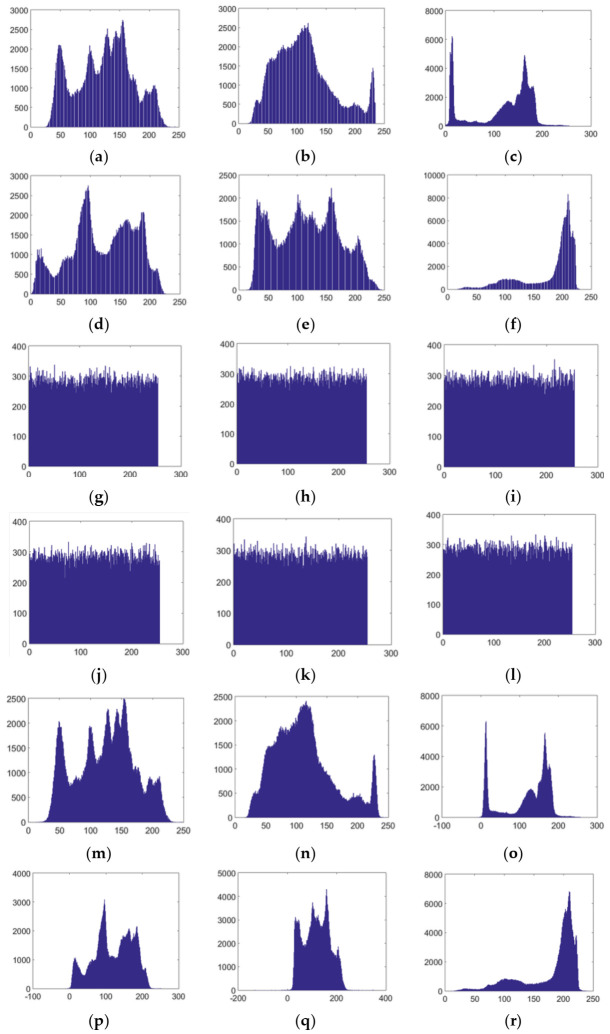
Histograms of (**a**–**f**) plain images Lena, Goldhill, Cameraman, Peppers, Barbara, Jet; (**g**–**l**) corresponding cipher images; (**m**–**r**) corresponding decrypted images.

A histogram is an effective index to evaluate the distribution of pixel values. With an effective image encryption scheme, the histogram of a cipher image should be evenly distributed, so as to effectively resist statistical attacks. In this experiment, the histograms of the images in Figure 4 were drawn, with results as shown in Figure 5, from which it can be seen that the pixels of cipher images are uniformly distributed, and are quite different from those of the original images, making it impossible to obtain useful information. The histograms of the reconstructed images are similar to those of the corresponding plain images, which indicates that the reconstruction effect of decrypted images is good. All in all, attackers can obtain no useful information about plain images through statistical attacks when the proposed scheme is used.

### 5.3. Sensitivity Analysis

Key sensitivity and plain sensitivity are important metrics to evaluate cryptosystems. Weak sensitivity enables the easy attack of a cryptosystem. We tested the key sensitivity and plain sensitivity of our scheme.

#### 5.3.1. Plain Sensitivity

In a differential attack, the attacker encrypts an original and modified plain image with the same key and determines the relationship between the plain and encrypted images by comparing the corresponding cipher images. To resist such attacks, an encryption algorithm should have strong plain sensitivity, i.e., two plain images with small differences should have significant differences after encryption. The main indicators to measure the difference between two images include number of pixels change rate (NPCR) and unified average changing intensity (UACI) [51], which respectively reflect the proportion of the number of different pixels to the size of the image and the average ratio of the differences between pixels at corresponding positions and 255. These are expressed as

(46) $$\mathrm{NPCR}\left(P_{1},P_{2}\right)\;=\frac{1}{\mathrm{MN}}\sum_{i=1}^{M}\sum_{j=1}^{N}\left|\mathrm{sgn}\left(P_{1}\left(i,j\right)-P_{2}\left(i,j\right)\right)\right|\;\times \;100\%$$

where sgn(·) is the sign function,

(47) $$\mathrm{sgn}\left(x\right)\;=\left\{\begin{array}{c} 1,x\;>0\\ 0,x\;=0\\ -1,x\;<0 \end{array}\right.$$

and

(48) $$\mathrm{UACI}\left(P_{1},P_{2}\right)\;=\frac{1}{\mathrm{MN}}\overset{M}{\underset{i=1}{\sum }}\overset{N}{\underset{j=1}{\sum }}\frac{\left|P_{1}\left(i,j\right)-P_{2}\left(i,j\right)\right|}{255-0}\times 100\%$$

The critical values of NPCR and UACI of the two random images were 99.6094% and 33.4635%, respectively. For NPCR, the more the critical value is exceeded, the stronger the plain sensitivity of the encryption scheme. For UACI, the closer to the critical value, the stronger the plain image sensitivity of the encryption scheme. In the experiment, one pixel in plain image $P_{1}$ was randomly selected and the change of its pixel value was set to 1 to obtain the modified plain image $P_{2}$. Cipher images $C_{1}$ and $C_{2}$ were obtained by encrypting plain images $P_{1}$ and $P_{2}$, respectively, with the same key, and NPCR and UACI were calculated, as shown in Table 12. The experimental results show that NPCR exceeded the critical value for all images except Peppers, and UACI was close to the critical value for all images. Hence, the proposed encryption scheme has a strong resistance to differential attacks.

#### 5.3.2. Key Sensitivity

**Figure 6:**
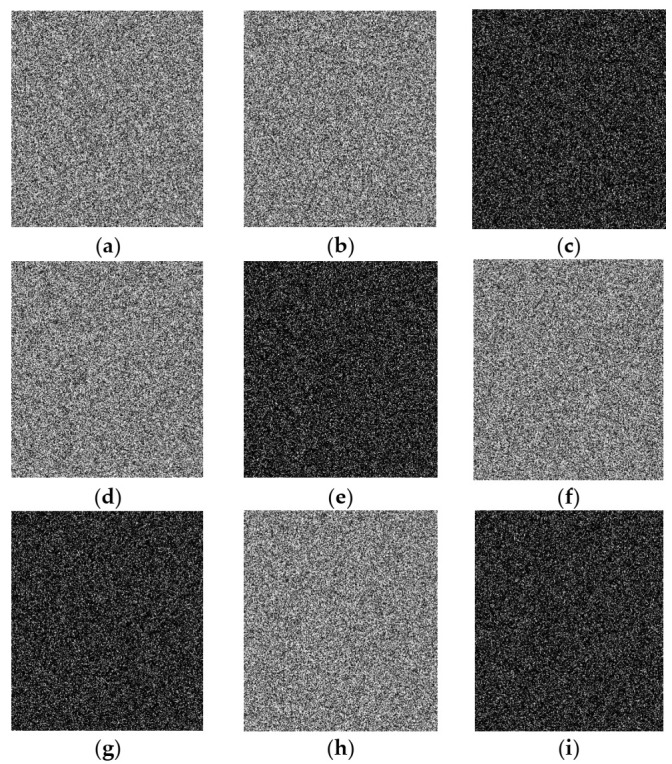
Experimental results of key sensitivity in encryption process: (**a**) cipher image using correct key K; (**b**,**d**,**f**,**h**) cipher images using wrong keys K1–K4, respectively; (**c**,**e**,**g**,**i**) differential images between (**a**,**b**), (**a**,**d**), (**a**,**f**), (**a**,**h**), respectively.

An encryption scheme should have high sensitivity to the secret key in both the encryption and decryption processes [9], i.e., a small change of the secret key should produce a completely different cipher image. Similarly, in decryption, the plain image should not be recovered by a decryption key differing slightly from the encryption key. We tested the key sensitivity from the aspects of sensitivity in both encryption and decryption. The initial values of the four chaotic systems used in this paper were changed slightly from $K\;=\;\left(x_{0},x_{00},y_{00},v_{0}\right)$ to $K1=\;\left(x_{0}+10^{-14},x_{00},y_{00},v_{0}\right)$, $K2=\;\left(x_{0},x_{00}+10^{-14},y_{00},v_{0}\right)$, $K3=\;\left(x_{0},x_{00},y_{00}+10^{-14},v_{0}\right)$, and $K4=\;\left(x_{0},x_{00},y_{00},v_{0}+10^{-14}\right)$. The Lena image was encrypted with the correct and wrong keys, with results as shown in Figure 6. From the experimental results, it can be seen that a small change in the key can cause a huge change in the cipher image but will not reveal information related to the plain image. To quantify the differences between cipher images obtained from the same plain image, NPCR and UACI were calculated using the correct and wrong keys to encrypt images, with results as shown in Table 13. It can be seen that NPCI and UACI are all close to the theoretical values, and more than 99.5% of pixels were modified, which implies that the encryption process is highly sensitive to the secret key.

**Figure 7:**
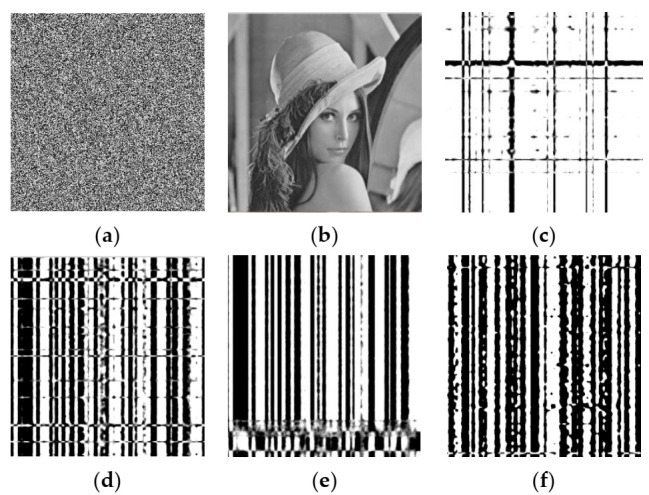
Experimental results of key sensitivity in decryption process: (**a**) cipher image using correct key K; (**b**–**f**) decrypted images of (**a**) obtained using K, K1, K2, K3, K4, respectively.

Next, a cipher image was decrypted with both the correct and wrong keys, with results as shown in Figure 7, from which it can be seen that only through the correct key can we get the correct decrypted image. Even a slight key change produces a visually meaningless image. Table 14 lists the values of NPCR from Figure 7b–f. When the key has a tiny change, more than 99.8% of pixels are altered, which indicates that the decryption process is highly sensitive to the secret key.

### 5.4. Correlation Coefficients

Strong correlations, close to 1, exist between the pixels of natural images. The correlation coefficient reflects the linear relationship between adjacent pixels and is an important index to evaluate image encryption schemes. For a good encryption algorithm, correlations between adjacent pixels of cipher images should be very weak, tending to zero, which means that the correlation between pixels is largely eliminated. The correlation coefficient of u and v is calculated as [52]

(49) $$\left\{\begin{array}{l} r_{\mathrm{xy}}=\frac{\mathrm{cov}\left(u,v\right)}{\sqrt{D\left(u\right)}\sqrt{D\left(v\right)}}\\ \mathrm{cov}\left(u,v\right)\;=\frac{1}{N}\overset{N}{\underset{i=1}{\sum }}\left(x_{i}-E\left(u\right)\right)\left(y_{i}-E\left(v\right)\right)\\ D\left(u\right)\;=\frac{1}{N}\overset{N}{\underset{i=1}{\sum }}{\left(u_{i}-E\left(u\right)\right)}^{2}\\ E\left(u\right)\;=\frac{1}{N}\overset{N}{\underset{i=1}{\sum }}u_{i} \end{array}\right.$$

where N is the number of adjacent pixel pairs from the image, and $\left(u_{i},v_{i}\right),i=1,2,\ldots ,N$ is the gray value of a pair.

**Figure 8:**
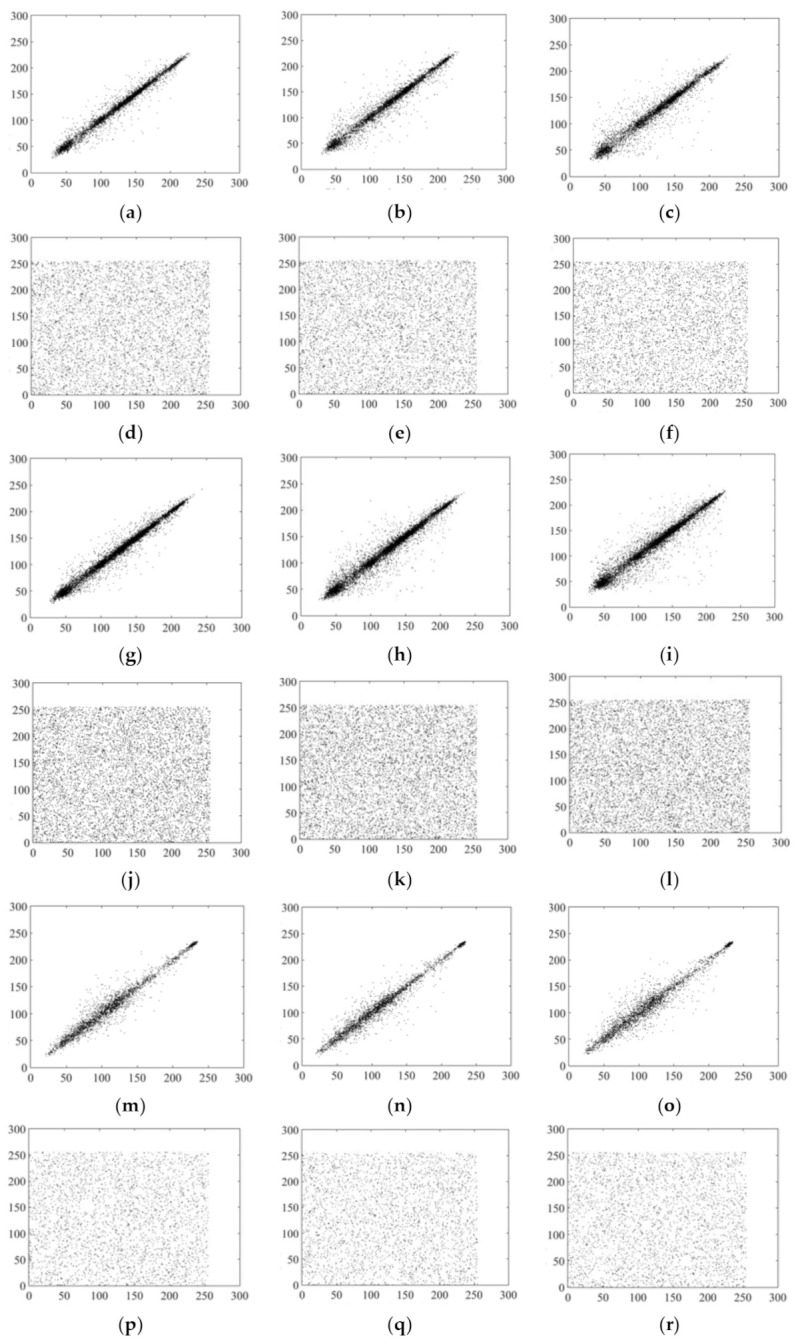

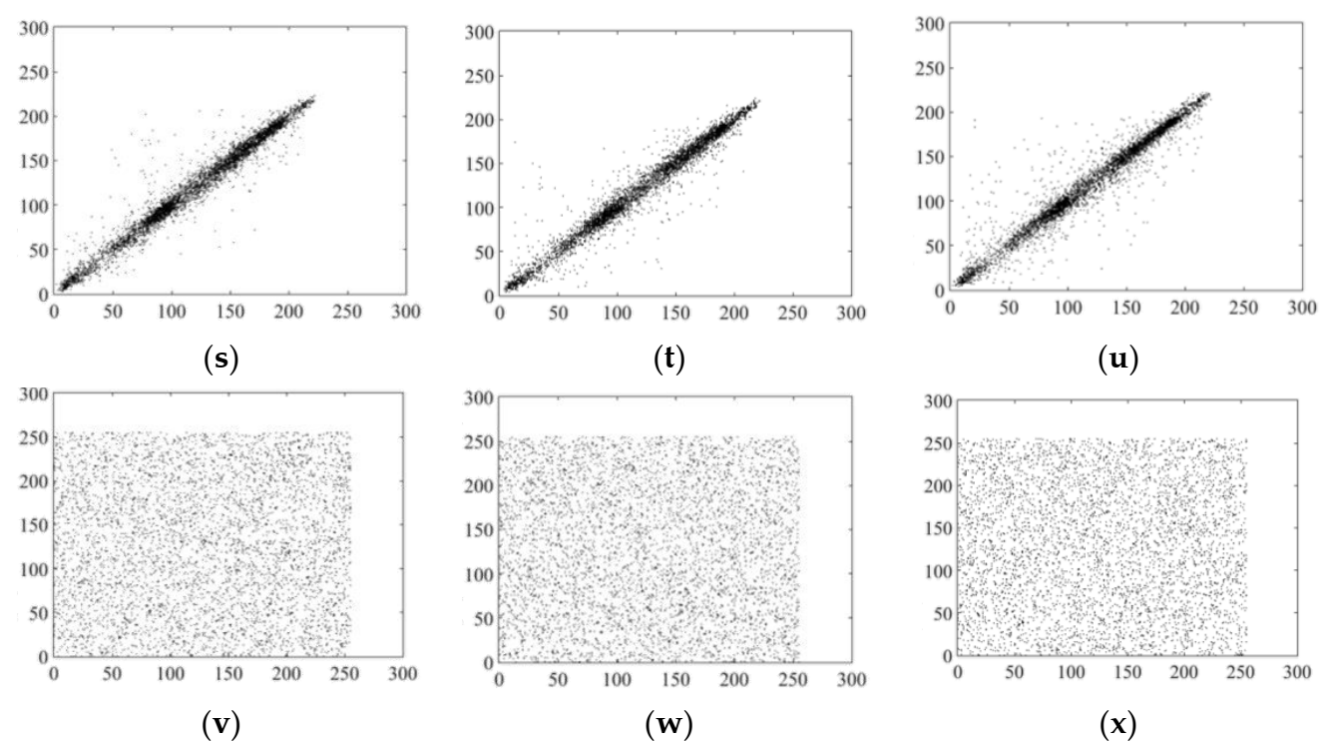
Histogram analysis. Horizontal, vertical, and diagonal pixel distributions of (**a**–**c**) 5000 pairs of adjacent pixels in plain image Lena at sampling rate of 0.75; (**d**–**f**) corresponding cipher image; (**g**–**i**) 8000 pairs of adjacent pixels in plain image Lena at sampling rate of 0.25; (**j**–**l**) corresponding cipher image; (**m**–**o**) plain image Goldhill; (**p**–**r**) corresponding cipher image; (**s**–**u**) plain image Peppers; (**v**–**x**) corresponding cipher image.

In this experiment, 512 × 512 images Lena, Goldhill, and Peppers were selected as test images, and 5000, 6000 or 8000 pairs of adjacent pixels were randomly selected from the cipher images in the horizontal, vertical and diagonal directions for calculation. To reduce randomness, each calculation was carried out 100 times, the final result was taken as the average value, and this was compared with other schemes. The experimental results are shown in Table 15, and show that the correlations between adjacent pixels of the cipher images were small. We also determined adjacent pixel distributions for plain and cipher images, with results as shown in Figure 8, showing that adjacent pixels of the plain images were basically linearly distributed. However, there were weak correlations between adjacent pixels of the compressed cipher images, with correlation coefficients close to 0, showing that the proposed scheme has a good encryption effect.

### 5.5. Information Entropy

Information entropy reflects the randomness of the distribution of image pixels and is calculated as [37]

(50) $$H\left(s\right)=-\overset{2^{n}-1}{\underset{i=0}{\sum }}p\left(s_{i}\right)\log_{2}p\left(i\right)$$

where $p\left(s_{i}\right)$ is the probability of the i-th pixel value $s_{i}$ of image p, and n is its total number of digits. For an 8-bit grayscale image, its cipher image should have information entropy near 8 bits. In this experiment, the 512 × 512 images Lena, Peppers, Cameraman, Barbara, and Jet were selected as test images. The experiment was carried out at sampling rates of 0.5 and 0.25, with results as shown in Table 16, from which it can be seen that the information entropies of cipher images were all greater than 7.99. The results show the encryption effect of the proposed scheme was better than that of comparison schemes [10,40,43], which indicates that our scheme has better randomness than other schemes based on compressed sensing. Besides, compared to the algorithm proposed by Brindha et al. [53], our scheme can achieve higher information entropy. It shows that although our lossy compression algorithm using compressed sensing cannot fully restore the original image from the cipher image, it can obtain more secure cipher images than the lossless compression algorithm. What is more, it can be seen that the cipher images generated from our encryption scheme have greater information entropies than those of schemes based on substitution box [14] and quantum key image [28] in most instances, which means our scheme is more resistant to entropy attack than some encryption schemes based on non-chaotic techniques.

### 5.6. Robustness Analysis

During the transmission process, cipher images can be contaminated by noises and data loss, making it hard to decrypt them and recover the corresponding plain images. In this experiment, noise and crop attacks were utilized to test the robustness of the proposed scheme.

#### 5.6.1. Noise Attack

**Figure 9:**
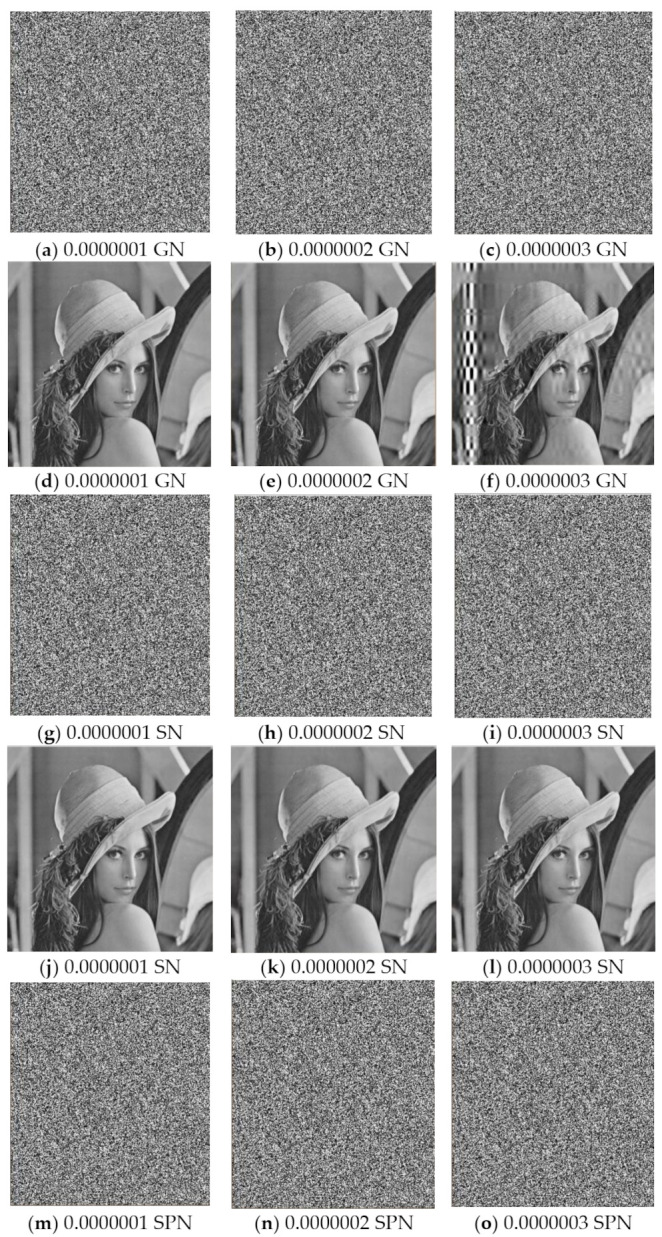

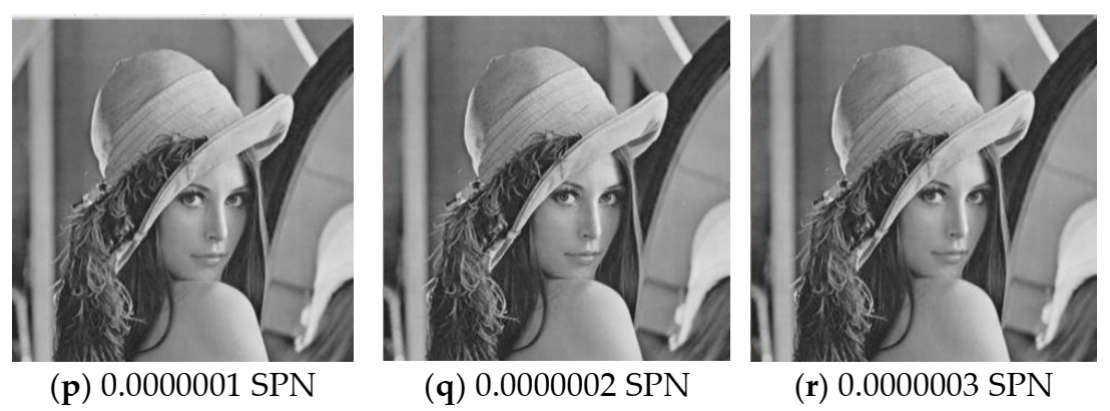
(**a**–**c**) Cipher images contaminated by Gaussian noise; (**d**–**f**) corresponding decrypted images; (**g**–**i**) cipher images contaminated by speckle noise; (**j**–**l**) corresponding decrypted images; (**m**–**o**) cipher images contaminated by salt-and-pepper noise; (**p**–**r**) corresponding decrypted images.

Common types of noise include Gaussian noise (GN), speckle noise (SN), and salt-and-pepper noise (SPN). To evaluate the robustness of the proposed scheme to noise attacks, Lena was encrypted as the test image, different levels of three noises were added, and decrypted images were obtained, with experimental results as shown in Figure 9 from which it can be seen that the proposed scheme has a stronger resistance to speckle noise and salt-and-pepper noise than Gaussian noise.

#### 5.6.2. Crop Attack

**Figure 10:**
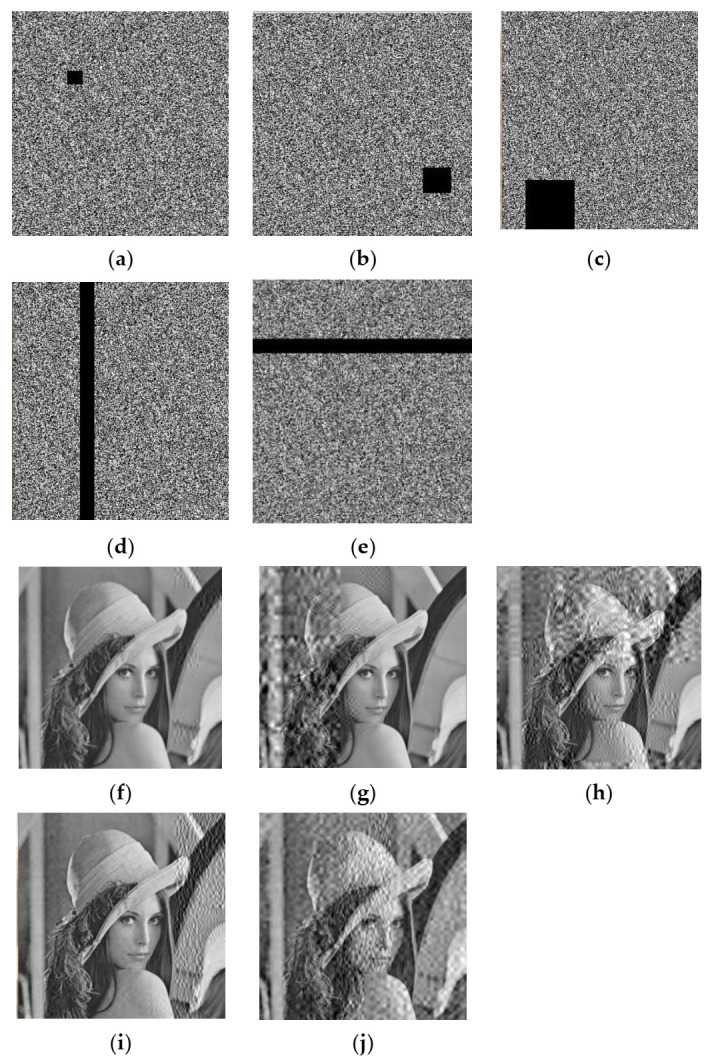
(**a**–**e**) Cipher images after cutting blocks of size 16 × 16, 32 × 32, 64 × 64, 288 × 16, 16 × 256, respectively; (**f**–**j**) corresponding decrypted images.

Different areas of cipher images were randomly selected after encrypting Lena, cutting image blocks of size 16×16, 32×32, 64×64, 288×16,16×256 to obtain cipher images with some data loss, along with corresponding decrypted images. The results are shown in Figure 10, and numerical quantization results of decrypted images are shown in Table 17. From the results, although a crop attack will worsen the visual effect of decrypted images, the proposed scheme still retains most information of the image, i.e., the scheme has a certain robustness to crop attacks.

## 6. Conclusions

An image encryption scheme based on multiscale block compressed sensing theory was proposed. In the scheme, considering that different information is carried by the low- and high-frequency coefficients of an image, different sampling rates were set for the low-and high-frequency coefficients of the image, so as to improve the reconstruction quality of decrypted images. Our scheme was experimentally compared with a scheme using the traditional compressed sensing theory and setting a sampling rate in each encryption process. Under the same compression ratio, the PSNR values between natural images and corresponding decrypted images of our scheme were better by more than 8 dB and were better by about 3–5 dB over a scheme that sets different sampling rates. Therefore, the proposed encryption scheme is suitable for natural image transmission with complex structures and large amounts of information. The safety of images is ensured, image information can be better preserved, and better visual effects can be obtained. With the combination of chaotic systems and a Markov model, the image is scrambled inside each coefficient matrix and is then scrambled among the coefficient matrices, and the encryption is completed by the strategies of independent and global diffusion. Experimental results show that the proposed scheme has a large key space, high plain sensitivity, and high key sensitivity in both the encryption and decryption processes. In particular, compared with an encryption scheme designed for problems of low entropy, the experimental scheme in this paper has a certain increase in information entropy values of most natural images, which shows the Markov probability model has certain advantages over traditional scrambling methods in simulating random processes. Therefore, the scheme has a certain guiding role for our future research work. At the same time, the scheme can effectively resist brute-force, differential, and statistical attacks. It also has a certain robustness to noise and crop attacks. However, high-intensity attacks cause large information loss, resulting in a poor visual effect. Hence, the design of a more robust encryption scheme warrants further study. Due to the use of multiscale block compressed sensing theory that better suits images, this scheme is suitable for mass image data transmission and can effectively save transmission space. To be honest, the times of the encryption and decryption processes in our scheme are long. Therefore, it remains to study the replacement of existing methods by more efficient chaotic systems and reconstruction methods while maintaining good encryption and decryption effects.

## Figures and Tables

**Table 1:** Transition probability matrix.

	$a_{1}$	$a_{2}$	…	$a_{j}$	…
$a_{1}$	$p_{11}$	$p_{12}$	…	$p_{1j}$	…
$a_{2}$	$p_{21}$	$p_{22}$	…	$p_{2j}$	…
⋮	⋮	⋮		⋮	
$a_{i}$	$p_{i1}$	$p_{i2}$	…	$p_{ij}$	…
⋮	⋮	⋮		⋮	

**Table 2:** State transition frequency matrix.

	po-od Number	ne-od Number	po-ev Number	ne-ev Number
po-od number	3696	3796	4249	3289
ne-od number	3833	3928	4409	3449
po-ev number	4217	4471	4937	3733
ne-ev number	3284	3424	3763	2962

**Table 3:** State transition probability matrix.

	po-od Number	ne-od Number	po-ev Number	ne-ev Number
po-od number	0.2459	0.2526	0.2827	0.2188
ne-od number	0.2454	0.2515	0.2823	0.2208
po-ev number	0.2429	0.2576	0.2844	0.2151
ne-ev number	0.2445	0.2549	0.2801	0.2205

**Table 4:** Rule of column vector scrambling.

	po-od Number	ne-od Number	po-ev Number	ne-ev Number
po-od number	Odd column↑$w_{1}$	Odd column↑$w_{3}$	Odd column↑$w_{2}$	Odd column↑$w_{4}$
ne-od number	Odd column↓$w_{1}$	Odd column↓$w_{3}$	Odd column↓$w_{2}$	Odd column↓$w_{4}$
po-ev number	Even column↑$w_{1}$	Even column↑$w_{3}$	Even column↑$w_{2}$	Even column↑$w_{4}$
ne-ev number	Even column↓$w_{1}$	Even column↓$w_{3}$	Even column↓$w_{2}$	Even column↓$w_{4}$

**Table 5:** PSNR (dB) of decrypted images at different sampling rates compared with another scheme.

Algorithm	Image	Sampling Rates						
		0.25	0.45	0.5	0.65	0.75	0.85	0.95
Ref. [10]	Lena	31.4240	32.9660	33.2299	33.8000	34.1313	34.5656	34.9347
Ours		34.9174	37.2453	37.6716	39.0365	40.0310	41.2019	42.7182
Ref. [10]	Peppers	30.6809	31.9825	32.1889	32.7692	33.1721	33.5154	33.9144
Ours		33.8452	35.9842	36.3212	37.4118	38.2535	39.2356	40.4900
Ref. [10]	Cameraman	30.4164	30.7728	31.2277	32.6649	34.2180	35.0159	35.4416
Ours		36.7108	39.5282	39.9194	40.7538	41.1572	41.4790	41.7297
Ref. [10]	Couple	30.1862	31.5430	31.9254	32.6734	32.8551	33.3367	33.7551
Ours		29.6688	32.7551	33.2811	35.0115	36.4607	38.3650	41.3051

**Table 6:** Comparison of decrypted image quality of similar scheme at the sampling rate of 0.5.

Algorithm	Image	PSNR(dB)
Ref. [48]	Lena	34.5560
Ours		37.6716
Ref. [48]	Cameraman	34.6995
Ours		39.9194
Ref. [48]	Peppers	31.5132
Ours		36.3212
Ref. [48]	Lake	29.2165
Ours		33.2254

**Table 7:** Influence of different wavelet bases on image reconstruction effect (PSNR: dB).

	**Image**	**Lena**	**Goldhill**	**Cameraman**	**Peppers**	**Barbara**	**Jet**
**Wavelet Bases**							
Symlets8		35.1252	30.7277	35.6823	33.5171	25.4683	32.9417
Haar		33.1509	30.3997	34.8881	29.6579	25.5130	29.8575
CDF9/7		35.0509	31.6262	36.8624	33.9987	25.2430	32.8437
	**Image**	**Mandril**	**Couple**	**Private**	**Blonde**	**Darkhair**	**Boat**
**Wavelet Bases**							
Symlets8		29.4729	29.9571	31.6857	30.2489	38.0362	30.8929
Haar		28.9758	29.5406	30.7943	29.6424	35.2409	30.4848
CDF9/7		29.8058	29.6773	31.7861	30.9151	38.6019	30.7819

**Table 8:** Runtime statistics of main processes at the sampling rate of 0.25.

Process	Chaotic Systems	Compression	Scrambling	Diffusion	Reconstruction
Time(s)	2.07138	0.002196	1.621437	0.994517	5.874532

**Table 9:** Runtime statistics of encryption process and decryption process at different sampling rates.

Sampling Rate	0.25	0.5	0.75
Encryption time(s)	5.380744	10.217306	12.060107
Decryption time(s)	12.175169	12.456957	11.085230

**Table 10:** Comparison results on time complexity of Encryption algorithm.

Algorithm	Time Complexity
Ours	$\Theta \left(5\times m\times n\right)$
Ref. [10]	$\Theta \left(5\times m\times n\right)$
Ref. [49]	$2\times \Theta \left(4\times m\times n\right)$
Ref. [50]	$\Theta \left(8\times m\times n\right)+\Theta \left(m\times n\right)$

**Table 11:** Key space comparison.

Scheme	Ours	Ref. [10]	Ref. [40]	Ref. [43]	Ref. [44]
Key space	$10^{112}$	>$10^{79}$	$10^{80}$	$10^{75}$	$2.56\times 10^{59}$

**Table 12:** Calculation results of NPCR and UACI.

Image	Lena	Baboon	Barbara	Boat	Goldhill	Peppers	Random Image
NPCR(%)	99.6202	99.6257	99.6162	99.6297	99.6446	99.6039	99.6094
UACI(%)	33.4682	33.4758	33.4320	33.4897	33.4698	33.4500	33.4635

**Table 13:** NPCR and UACI of cipher images with the correct key and different wrong keys.

Secret Keys	K and K1	K and K2	K and K3	K and K4
NPCR (%)	99.5985	99.6053	99.6243	99.6134
UACI (%)	33.4227	33.5085	33.4998	33.3888

**Table 14:** NPCR of decrypted images with the correct key and different wrong keys.

Secret Keys	K and K1	K and K2	K and K3	K and K4
NPCR (%)	99.9298	99.8577	99.9943	99.9981

**Table 15:** Correlation coefficients of adjacent pixels in cipher images.

Algorithm	Image	Horizontal	Vertical	Diagonal
Ref. [10]	Lena	−0.0029	0.0058	−0.0025
Ours		−0.0049	−0.0036	0.0002
Ref. [44]	Lena	−0.0022	0.0023	0.0034
Ours		0.0074	0.0015	0.0010
Ref. [53]	Lena	0.0020	0.0033	0.0005
Ref. [14]		0.0081	0.0065	0.0182
Ours		0.0002	−0.0021	0.0037
Ref. [43]	Goldhill	0.0062	−0.0107	0.0052
Ours		−0.0039	−0.0047	0.0003
Ref. [10]	Peppers	−0.00072	−0.0155	0.00036
Ref. [14]		0.0082	0.0002	0.0088
Ref. [28]		0.01484	−0.1164	−0.0023
Ours		−0.0004	−0.0017	0.0053

**Table 16:** Comparison of information entropy of cipher images.

Image	Ref. [10]	Ref. [40]	Ref. [43]	Ref. [53]	Ref. [14]	Ref. [28]	Ours
Lena	7.9986	7.9575		7.9973	7.9022		7.9987
Cameraman	7.9987	7.9853					7.9988
Peppers	7.9986			7.9975	7.9513	7.9938	7.9987
Couple	7.9987						7.9981
Barbara			7.9970	7.9975			7.9974
Jet			7.9970			7.9978	7.9975

**Table 17:** Influence of different data loss on image reconstruction effect (PSNR).

Data Loss	0	16 × 16	32 × 32	64 × 64	288 × 16	16 × 256
PSNR (dB)	35.0509	32.0300	23.3382	20.8193	24.2573	11.4978

## Data Availability

Data are available upon reasonable request. Additional data (beyond those included in the main text) are available from the corresponding author upon request. The data are not publicly available due to [the data also forms part of an ongoing study].
